# Generating functional plasmid origins with OriGen

**DOI:** 10.1093/nar/gkaf1198

**Published:** 2025-11-29

**Authors:** Jamie Irvine, Jonathan N V Martinson, Jigyasa Arora, Sarah I Hasham, Jaymin R Patel, Emily T Qi, Brady F Cress, Benjamin E Rubin

**Affiliations:** Innovative Genomics Institute, University of California, Berkeley, CA 94720, United States; Innovative Genomics Institute, University of California, Berkeley, CA 94720, United States; Innovative Genomics Institute, University of California, Berkeley, CA 94720, United States; Innovative Genomics Institute, University of California, Berkeley, CA 94720, United States; Innovative Genomics Institute, University of California, Berkeley, CA 94720, United States; Innovative Genomics Institute, University of California, Berkeley, CA 94720, United States; Innovative Genomics Institute, University of California, Berkeley, CA 94720, United States; Innovative Genomics Institute, University of California, Berkeley, CA 94720, United States

## Abstract

While generative artificial intelligence has shown promise for biological design, no computational system has yet created sequences proven capable of replication. Focusing on plasmids as minimal replicating systems, we develop OriGen, a language model that generates novel plasmid origins of replication while maintaining essential functional elements. We experimentally validate OriGen’s ability to create active origins in *Escherichia coli* that diverge from existing wild types, demonstrating the model’s capacity to capture the complex and often enigmatic mechanisms of biological replication. Further, we directed OriGen to produce functional origins that avoid common restriction sites, demonstrating the model’s potential for automated controllable design of vectors with desirable properties.

## Graphical Abstract

**Graphical Abstract:**
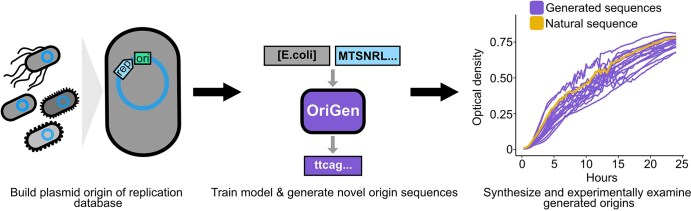

## Introduction

In recent years, artificial intelligence (AI) has transformed how we study biological systems, with tools such as AlphaFold revealing fundamental insights about molecules and their interactions [1]. While AI has been invaluable for these descriptive tasks, its potential for biological design remains largely untapped. Current bioengineering approaches rely heavily on manual design and laborious trial-and-error, confining our capabilities to a handful of model systems [2, 3]. Recent advances in generative modeling, exemplified by groundbreaking achievements in natural language processing [4, 5], suggest a promising path forward. These tools can overcome current limitations by rapidly creating novel sequences for user-defined functions, efficiently exploring vast regions of sequence space that remain otherwise inaccessible. Combined with ever-decreasing synthesis costs, this approach has the potential to dramatically accelerate bioengineering, enabling bespoke solutions for the wide diversity of biological design challenges.

Recent work has shown promising steps toward machine-generated biological sequences. Protein generation models like ProGen [6] and ProtGPT2 [7] have demonstrated the ability to create novel functional proteins across a range of structural families. The field has also expanded into DNA sequence generation, with large language models like GenSLMs [8] and Evo [9] showing increasing capability to generate both coding and non-coding regions in a diversity of settings. But despite growing investment in these DNA foundation models, they are still in their infancy, and have yet to demonstrate mastery of the full spectrum of sequences and functions. Notably, plasmids have received surprisingly little attention from generation efforts, despite being crucial for the transfer and evolution of new genes and essential tools for biological engineering. While there has been some recent work generating plasmid and phage genomes *in silico*, the outputs remain untested in biological systems [10, 11]. In particular, no study has experimentally validated AI-generated sequences capable of plasmid replication—the only essential component of a plasmid, and a fundamental aspect of life itself.

Here we address this gap by developing OriGen, a language model specifically trained to generate replicons—the minimal genetic units required for DNA replication. We assemble a comprehensive database of plasmid replicons and their host associations, then use this to train a model that can generate novel replication sequences. OriGen is validated by synthesizing generated origins of replication (*oriVs*) and testing their ability to replicate in *Escherichia coli* and characterizing the resulting bacterial growth dynamics and plasmid copy numbers. Additionally, we show proof of principle for constraint-guided generation, with OriGen producing origins that avoid specific restriction sites while maintaining replication function, suggesting potential for practical bioengineering applications. This work represents the first experimentally validated AI-generated biological sequences capable of replication.

## Materials & methods

### Training data generation

To expand the plasmid replicon database, the plasmids in the PLSDB [12] (version 2023_11_23_v2) and IMG/PR [13] datasets were annotated using Prokka [14] (version 1.14.6) and Prodigal [15] (version 2.6.3) software. The non-coding regions were defined from these annotations using the BEDTools suite software toolkit [16] (version 2.31.1). All the protein sequences were annotated against the PFAM database [17] (version 33.1) using hmmsearch (version 3.4, http://hmmer.org/). The Rep proteins were annotated by their PFAM domains. These annotations were supplemented with custom Rep HMMs generated by the plaSquid software [18] and BLASTn against the MOB-suite Replication protein database [19].

The origins were annotated by BLASTn against the DoriC database [20] (version 12.0) and filtered for 80% identity and 80% coverage. To prevent misclassification, BLASTn-selected origins were further filtered based on their proximity to Rep proteins.

Host assignments were inferred from the isolation source and CRISPR spacer matching data within PLSDB and IMG/PR [12, 13].

The incompatibility group typing was performed using the MOB-suite package. The group types associated with the replication initiation proteins were extracted, and the top 12 most frequent incompatibility groups were selected for analyses.

### Model training

Training and validation splits were created by first clustering *oriV* sequences from the combined DoriC and PLSDB datasets using CD-HIT (v4.8.1) with a 90% sequence identity threshold. The resulting clusters were then randomly split 75/25 for training and validation, with the validation set used for hyperparameter tuning and early stopping. The IMG/PR dataset was reserved as a separate held-out test set, after excluding any *oriVs* with greater than 95% similarity (BLAST pident > 95%, qcovs > 95%) to any *oriV* in our training split.

Input sequences were tokenized as discrete units: bacterial host species names as single tokens, amino acids for Rep proteins, and individual nucleotides for *oriV* sequences (using lowercase to avoid collisions with amino acid letters). Special tokens handled cases of unknown or unannotated species and the separation of multiple Rep proteins when present. We chose amino acid-level tokenization for Rep proteins primarily to reduce sequence lengths within our maximum context window of 1500 tokens. The complete vocabulary contained 145 tokens.

OriGen uses a decoder-only transformer architecture with 12 layers and 12 attention heads per layer (embedding dimension 768), totaling approximately 86M parameters. The model was trained using the AdamW optimizer with standard dropout rates (0.1) and weight decay (0.1). Training used mixed precision and ran on a single NVIDIA RTX A5000 GPU, with early stopping based on validation loss.

### Model evaluation

To enable direct comparison between OriGen and Evo (8k base model), which uses single-nucleotide tokenization on contiguous DNA sequences, we adapted our test sequences accordingly. For each plasmid in the IMG/PR dataset, we extracted the contiguous DNA region containing both the Rep gene and its adjacent *oriV*. Since our evaluation focuses on *oriV* prediction conditioned on Rep sequence, we took the reverse complement of sequences where the Rep gene followed the *oriV*, ensuring Rep always preceded *oriV* while maintaining natural sequence contexts. Test sequences were filtered to exclude any with greater than 95% similarity to sequences in our training data (BLAST parameters: pident > 95%, qcovs > 95%). Since Evo’s train/test splits are not currently published, the majority of sequences in this evaluation were likely part of Evo’s training data. Prediction accuracy was calculated as the percentage of correctly predicted nucleotides across all test sequences, with 95% confidence intervals estimated through bootstrapping (n = 1000 resamples).

### Model embeddings

Per-sequence model embeddings were computed as the mean of the token embeddings for each token in the sequence. For visual analysis, the embeddings were dimensionally reduced using t-Distributed Stochastic Neighbor Embedding (t-SNE) in R using the ggplot2 and Rtsne packages.

### Specialized models for incompatibility groups

We selected the ten most frequent incompatibility groups in our training dataset and identified the corresponding sequences in the validation split and IMG/PR test set (Supplemental Fig. 2). For each group, we trained a specialized OriGen model using the same architecture and hyperparameters as the full model, with early stopping based on performance on the group-specific validation split.

Evaluation was performed by testing each specialized model on the subset of IMG/PR corresponding to its incompatibility group, and comparing per-nucleotide accuracy of the origins against the full OriGen model on the same held-out data. Prediction accuracy was reported as the percentage of correctly predicted nucleotides, with 95% confidence intervals estimated by bootstrapping (n = 1000 resamples).

### Model generations

For the analysis of unprompted replicon generation, sequences were sampled using nucleus sampling (top-p = 0.95) with temperature 1.0. For experimental validation, both replicon types were conditioned with a starting *E. coli* token. The ColE1 examples were further conditioned with the first 20 nucleotides from a ColE1 *oriV* from the validation split (with no amino acid tokens). The Rep3 sequences were conditioned with the amino acid sequence from the Rep gene identified in the chosen plasmid. These generations used top-k sampling (k = 4) with temperature 1.0. In both cases, generation proceeded until the model produced a stop token or reached the maximum sequence length.

### In-silico validation

To compare OriGen-generated sequences with wild-type and random sequences, we generated 1000 unprompted sequences from OriGen and extracted their *oriVs*. We compared these with 1000 wild-type *oriVs* randomly sampled from the training data. As a baseline, 1000 random nucleotide sequences were generated, each of length 357, the median *oriV* length in the training data.

The occurrence of known conserved motifs in *oriVs* was searched using FIMO software from the MEME suite [21]. These motifs were identified via a literature search of known motifs in the *oriVs*, such as DnaA boxes and iterons. The annotated motifs were filtered based on a log-likelihood score greater than 1.0 and the presence of all motifs within the region. AT content was calculated for each sequence using an in-house Python script. Minimum free energy (MFE) was calculated on a sliding window of 100 base pairs using RNAfold (version 2.5.1) from the ViennaRNA package [22]. The sliding window was created using Seqkit [23] (version 2.9.0). Mean MFE scores were calculated and plotted for each sequence. All sequences were aligned using MAFFT [24] (version 7.505), and a phylogenetic tree was constructed using FastTree with the GTR algorithm [25]. The tree was visualized in iTOL [26].

### Sequence similarity and matched random mutants

To experimentally test generated sequences with varying levels of novelty, we quantified sequence similarity using global alignment (Needleman–Wunsch algorithm, implemented via EMBOSS needle with gap_open = 10.0 and gap_extend = 0.5). For each generated sequence, we first identified candidate wild-type matches by BLAST search against our training data, then performed global alignment with the top BLAST hit to calculate a final similarity score (Supplemental Table 1).

To create matched random mutants as controls, we analyzed these alignments to determine the number of insertions, deletions, and point mutations between each generated sequence and its closest wild-type match. We then created control sequences by applying the same number of each mutation type (e.g. insertion, deletion, and SNP) at random positions in the wild-type sequence. Due to the interaction between mutations, global alignment of these random controls back to their wild-type sequences sometimes yielded different optimal alignments, resulting in similarity scores that could be slightly higher or lower than their model-generated counterparts.

### Analysis of functional characteristics

RNAI and RNAII regions of the tested ColE1 replicons were annotated using HMM profiles from a previous publication [27]. The longest aligned RNAI region was extracted using the samtools faidx command [28]. RNAI secondary structures were predicted for the extracted RNAI sequences using the RNAfold WebServer [22]. Conserved motifs in experimentally tested Rep3-associated *oriVs* were identified using the FIMO software from the MEME suite [21] as described above.

### Bacterial strains and culture conditions

Two bacterial strains were used in this study. Transformax EC100D *pir*+ (Epicenter, Cat. No. 75927-934) has the genotype *F⁻ mcrA Δ(mrr-hsdRMS-mcrBC) φ80dlacZΔM15 ΔlacX74 recA1 endA1 araD139 Δ(ara, leu)7697 galU galK λ⁻ rpsL (Str^R^) nupG pir⁺(DHFR)*. This strain was selected for its ability to maintain and propagate plasmids requiring the *pir* gene for replication, such as suicide plasmids with *R6K* origins. NEB 10-Beta (New England Biolabs, Cat. No. C3020K) has the genotype *Δ(ara-leu)7697 araD139 fhuA ΔlacX74 galK16 galE15 e14⁻ φ80dlacZΔM15 recA1 relA1 endA1 nupG rpsL (Str^R^) rph spoT1 Δ(mrr-hsdRMS-mcrBC)* and was chosen for its high transformation efficiency and lack of *pir*. Bacterial cultures were grown in lysogeny broth (LB; RPI, Cat. No. L24060-5000.0) supplemented with carbenicillin (100 µg/mL; Sigma–Aldrich, Cat. No. C1389) at 37°C with shaking at 200 rpm. For solid media, lysogeny broth containing 2% agar (BD, Cat. No. 214010) was used.

### Plasmid construction

The R6K suicide vector backbone used in this study was adapted from a previously developed part plasmid [29]. All PCRs were performed using Q5® High-Fidelity 2X Master Mix (New England Biolabs, Cat. No. M0492L) following the manufacturer’s instructions.

To insert the ColE1 origins, BsaI Golden Gate recognition sites were added to an in-house R6K vector via PCR amplification using oligonucleotides oJNVM0070 and oJNVM0073 (Extended Supplementary Data S1 and Supplementary Data S2). PCR amplicons were gel purified with the Zymoclean Gel DNA Recovery Kit (Zymo Research, Cat. No. D4002). ColE1 origins were synthesized as gene fragments (Twist Biosciences) containing BsaI sites and complementary overhangs (Extended Supplementary Data S3).

For the construction of the rep-associated origin vector, Gibson assembly was used to clone the Rep gene from a 4076 bp plasmid (NCBI reference sequence NZ_CP128885.1) hosted in an *E. coli* strain TUM14759 under the control of a weak promoter (BBa_J23114) and a ribosomal binding site designed with the “Control Translation” function on denovodna.com at a target translation efficiency rate of 50 000 a.u. [30]. Additionally, a SapI Golden Gate dropout cassette was inserted immediately downstream of the Rep gene, providing a site for the insertion of replication origins (Extended Supplementary Data S2). DNA fragments encoding the promoter, RBS, Rep gene, and dropout cassette were synthesized as a single gene fragment (Twist Biosciences) with overhangs homologous to the R6K vector.

Initial experiments indicated that wild-type origins of replication functioned only in the presence of a P5R mutation in the *rep3* gene. This mutation was introduced into the Rep dropout cassette vector using site-directed mutagenesis.

A plasmid containing both a fragment of the gene *dxs* and *bla* was generated with Gibson assembly to serve as a calibrator for the plasmid copy number qPCR reactions.

Golden Gate assemblies were performed using type IIS restriction enzymes BsaI-HF®v2 (New England Biolabs, Cat. No. R3733S) for ColE1 origins and SapI (New England Biolabs, Cat. No. R0569S) for rep-associated origins. Each reaction was performed with a final concentration of 4 nM vector and insert.

Gibson assemblies were performed using NEBuilder® HiFi DNA Assembly Master Mix (New England Biolabs, Cat. No. E2621L) following the manufacturer’s instructions.

### Transformation and screening of plasmids

Assemblies were transformed into either NEB 10-Beta or EC100D *pir *+ cells using electroporation with 0.1 cm Pulser/MicroPulser Electroporation Cuvettes (Bio-Rad, Cat. No. 1652083). Following electroporation, cells were recovered in SOC media (Invitrogen, Cat. No. 15544034) at 37°C for 30 min under static conditions. Recovered cells were then centrifuged at 3000 × g for 3 min, the supernatant was removed, and the entire cell pellet was streaked onto selective media plates.

Well-separated transformant colonies were picked and inoculated into 5 mL of lysogeny broth containing carbenicillin and incubated overnight at 37°C with shaking. Freezer stocks were prepared in LB containing 10% glycerol (Sigma–Aldrich, Cat. No. G33-1).

Plasmid DNA was extracted using the QIAprep Spin Miniprep Kit (Qiagen, Cat. No. 27106) and eluted with 30 µL molecular-grade water. DNA concentrations were quantified using a NanoDrop 2000 spectrophotometer, and plasmids were submitted for Oxford Nanopore plasmid sequencing (Plasmidsaurus). Assembled plasmid DNA sequences were aligned to reference sequences using SnapGene (version 8.0.1; Extended Supplementary Data S4).

### Growth curve analysis

For each origin, single colonies were inoculated in triplicate (one colony per well) into 500 µL LB broth supplemented with carbenicillin (100 µg/mL) in deep-well plates. Cultures were grown overnight at 37°C with shaking (200 RPM). The following day, cultures were adjusted to an OD_600_ of 0.2 and diluted 1:100 into clear, flat-bottom 96-well plates (CytoOne, Cat. No. CC7682-7596) containing LB broth with carbenicillin (100 µg/mL) in a final volume of 200 µL per well. Plates were incubated at 37°C with orbital shaking (282 cycles per minute with a 3 mm orbit), and OD_600_ measurements were recorded every 15 min for 1 day using an Agilent BioTek Synergy H1. Growth curve data were processed and analyzed in R v4.4.0 using the tidyverse and gcplyr packages. Raw optical density readings were blank corrected, and growth parameters were estimated using gcplyr functions [31]. Visualization and statistical analyses were performed in R using ggplot2 [32], emmeans [33], and broom. Maximum growth rates were compared across sequence similarity levels within each origin type using one-way ANOVA, followed by Tukey’s post-hoc test (⍺ = 0.05).

### Plasmid relative copy number evaluation

Cells were grown in LB broth supplemented with carbenicillin (100 µg/mL) with shaking under the same conditions described above for the growth curve analysis. Following growth, cultures were normalized to an OD_600_ of 0.2 and diluted 1:10 in molecular-grade water to a final volume of 100 µL. Cells were then lysed by incubating for 10 min at 100°C. Relative plasmid copy number was determined by quantitative PCR following the method of Lee et al. (2006). Briefly, primers (Extended Supplementary Data S1) targeting *bla* (plasmid target) and *dxs* (chromosomal reference) were used in reactions prepared with SsoAdvanced Universal SYBR Green Supermix (Bio-Rad, Cat. No. 1725270) according to the manufacturer’s instructions, and run on a QuantStudio™ 3 Real-Time PCR System (Thermo Fisher Scientific). Relative copy number was calculated using the ∆∆Ct method. A calibrator plasmid containing single copies of both the *bla* and *dxs* references was constructed to account for PCR bias (Extended Supplementary Data S2). Ct values were generated on the Thermo Fisher Connect Platform, then exported to R v4.4.0 for analysis. Relative copy number was calculated using the ∆∆Ct method implemented in custom R scripts [34]. For a subset of origins, technical artifacts (air bubbles in wells) compromised measurements; in these cases, all three biological replicates were repeated.

### Plasmid stability

For each origin, cells were grown in LB broth with carbenicillin (100 µg/mL) under the same conditions described above for the growth curve and plasmid copy number assays. Overnight cultures were adjusted to an OD_600_ of 0.2 and diluted 1:100 into 96-well plates (CytoOne, Cat. No. CC7682-7596) containing either LB broth (no antibiotic) or LB broth with carbenicillin (100 µg/mL) in a final volume of 200 µL per well. The plates were incubated with shaking (200 RPM) for 24 h. Cells were serially diluted in PBS and plated as 5 µL spots onto LB agar with and without carbenicillin (100µg/mL). The frequency of cells carrying the plasmid was calculated by dividing the number of carbenicillin-resistant cells by the total number of cells that grew on LB.

### Pooled plasmid fitness assay

For each ColE1 origin variant, three separate biological replicate cultures were grown overnight with antibiotic selection. The following day, each overnight culture was washed and diluted into fresh LB to a standardized OD_600_ of 0.2. To create the experimental populations, the replicates were pooled into three distinct mixtures. Each pool was created by combining a single, unique biological replicate from each of the different origin variants. Immediately after pooling, a sample was taken from each mixture to serve as the time point zero (T0) control. These T0 samples were centrifuged, the supernatant was decanted, and the cell pellets were stored at −20°C. The competitive growth assay was initiated by diluting each of the three pooled mixtures 1:100 into 5 mL of LB, with one set of cultures containing 100 µg/mL carbenicillin and a parallel set containing no antibiotic. These cultures were grown for 24 h at 37°C with shaking at 200 RPM. After 24 h, the cultures were passaged by transferring a 1:100 dilution into fresh media corresponding to their respective antibiotic condition, corresponding to approximately 6.6 generations of growth per passage. A sample from the remaining culture was collected by centrifugation, and the cell pellet was frozen at −20°C. This process of passaging and sampling was repeated for a total of three passages over 3 days.

At the end of the experiment, plasmid DNA was extracted from the thawed cell pellets using the QIAprep Spin Miniprep Kit (Qiagen, Cat. No. 27106) and eluted with 30 µL molecular-grade water. The extracted plasmid DNA was normalized to a concentration of 2.5 ng/µL. This DNA was used as a template to amplify the ColE1 origin region using primers that flanked the origin (oJNVM0074 and oJNVM0075). In 50 µL reactions, PCR was carried out with Q5® High-Fidelity 2X Master Mix (New England Biolabs, Cat. No. M0492L) using the following program: 98°C for 30 s; 25 cycles of 98°C for 10 s, 63°C for 20 s, and 72°C for 30 s; and a final extension at 72°C for 2 min. After amplification, the amplicons were treated with DpnI (New England Biolabs, Cat. No. R0176S) to digest the remaining plasmid template and subsequently purified with the QIAquick PCR Purification Kit (Qiagen, Cat. No. 28104) following the manufacturer’s instructions. Purified amplicons were normalized to 10 ng/µL and submitted for Oxford Nanopore sequencing through Plasmidsaurus using the Premium PCR Sequencing option, which provides full-length, end-to-end reads using the v14 library prep chemistry in R10.4.1 flow cells. We received ∼4700 reads per sample.

To quantify the abundance of each origin variant, sequencing reads were aligned to a reference index of the origin sequences using plasmap, a custom wrapper script utilizing minimap2 and Samtools. Alignments were generated with the map-ont preset in minimap2. The resulting SAM output was then processed with Samtools to filter for primary alignments with a mapping quality (MAPQ) of 20 or greater, while simultaneously removing secondary, supplementary, and unmapped reads. Final counts for each origin variant were generated by tabulating the reference sequence name for each of the remaining alignments.

All subsequent data analysis was performed with custom scripts in R. The raw read counts for each variant were first used to calculate its relative frequency within each sample’s total read population. A pseudocount of 1 × 10^-6^ was added to all counts to prevent division by zero. The relative fitness (*W*) of each variant at a given time point (*t_x_*) was calculated relative to its starting frequency at time zero (*t_0_*) using the formula:


\begin{eqnarray*}
W = lo{{g}_2}\left( {{{f}_{tx}}/{{f}_{t0}}} \right)
\end{eqnarray*}

where *f* is the frequency of the variant in each sample. One origin (87%) was excluded from downstream analyses due to a point mutation in the primer binding region that interfered with amplification.

### Constraint-guided generation

The list of restriction enzyme recognition sites to avoid was compiled from the REBASE restriction–modification system database (New England Biolabs) via FTP in June 2025. We parsed the database to identify and quantify type II restriction–modification recognition sites in *E. coli*, retaining the 20 most frequently occurring motifs. Sites that were fewer than three nucleotides long, or that contained more than five degenerate positions (wildcards), were excluded for simplicity, yielding the following twelve motifs to avoid in sequence generations: GATC, CCWGG, GGTCTC, GAAABCC, GRGCYC, CCGCGG, CTGCAG, CCNGG, GGYRCC, CRARCAG, GAATTC, and CCTNAGG.

For each selected site, all degenerate bases were expanded to their full set of nucleotide variants, and the reverse complement of each sequence was added. The resulting expanded list was provided to the HuggingFace Transformers library as bad_words_ids during generation, ensuring that OriGen did not produce sequences containing any of the targeted motifs. Beam search was used during generation to increase the diversity of viable outputs under these constraints.

ColE1-type origins were generated by prompting OriGen with the first 20 nucleotides of a canonical wild-type ColE1 origin, consistent with other experiments in this study. Because this prefix itself contained two recognition sites from the avoidance list, the affected subsequences were replaced post-hoc with the model’s highest-likelihood infill predictions, ensuring that the full sequence was free of restriction sites from the list.

For baseline sequences, the same canonical ColE1 origin used for the seed prompt was scanned for occurrences of the selected restriction sites. For each site, one nucleotide was randomly chosen and substituted with a random different nucleotide. This process was repeated iteratively until no recognition sites remained in the sequence, yielding wild-type-derived control origins that avoided the targeted sites by the minimal number of substitutions.

## Results

### Model training and *in silico* validation

**Figure 1.**
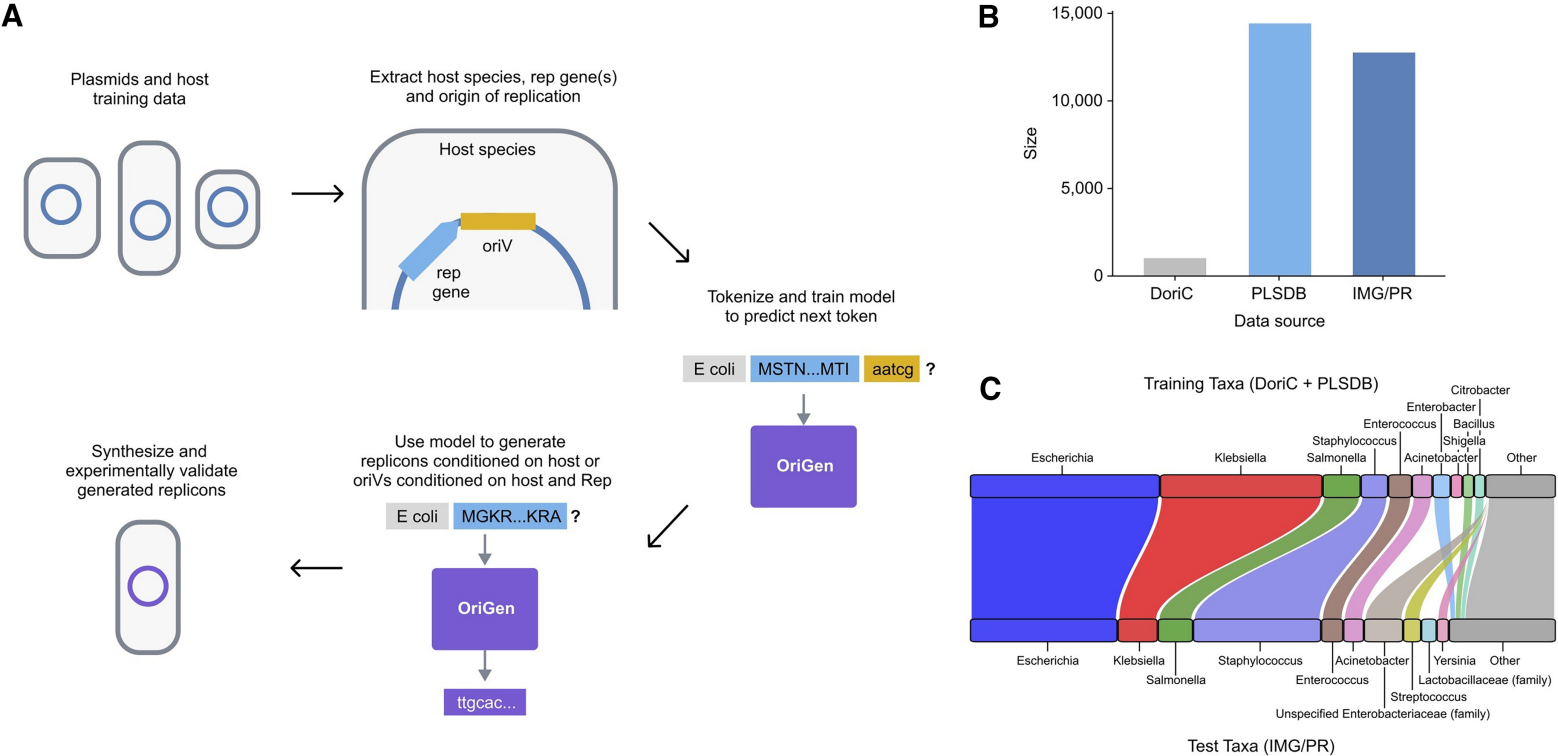
Development of OriGen for plasmid origin generation. (**A**) Schematic of the OriGen training, generation, and validation pipeline. (**B**) Replicon data extracted from different plasmid datasets. (**C**) Distribution of genera present in DoriC + PLSDB versus IMG/PR. In some instances, host taxonomy is only known at a higher taxonomic rank, indicated in parentheses.

OriGen is an autoregressive language model capable of generating host-dependent plasmid replicons, which consist of an *oriV* and Replication initiation (Rep) protein(s) (Fig. 1A). To train this model, we first assembled a comprehensive dataset of *oriVs* and Reps from plasmids, along with their bacterial hosts. This presented a significant challenge, as *oriVs* are poorly characterized and difficult to identify computationally. The only dedicated database of plasmid *oriVs*, DoriC [20], provides carefully curated sequences, but contains relatively few examples. To expand this dataset, we developed a BLAST-based pipeline to identify putative *oriVs* in two larger plasmid databases: PLSDB [12] and IMG/PR [13]. By aligning the non-coding region of the plasmids from these databases against known *oriVs* from DoriC, this pipeline added 14 424 unique newly annotated replicons from PLSDB and 12 769 from IMG/PR to the 1023 examples in DoriC (Fig. 1B). Associated Rep genes were identified using a Rep-specific HMM search [17, 18] and protein database match [19]. The resulting dataset is biased toward the representation of DoriC, but it still contains significant diversity in host genera and incompatibility groups (Supplemental Fig. 1). It is the largest known collection of annotated plasmid replicon sequences to date.

OriGen was trained on this replicon dataset. For each plasmid, we extracted and tokenized three components into a single sequence: the bacterial host species, followed by the amino acid sequence(s) of the Rep protein(s) (when present), followed by the *oriV* nucleotide sequence. To ensure robust evaluation, we combined the DoriC and PLSDB datasets and split them into training and validation splits based on *oriV* sequence similarity, and we reserved the IMG/PR dataset as a held-out test set to evaluate domain-transfer performance across markedly different data sources (Fig. 1C). Once trained, OriGen can generate new replicon sequences from scratch, replicons conditioned for a desired host species compatibility, or *oriVs* conditioned on a given Rep gene.

**Figure 2.**
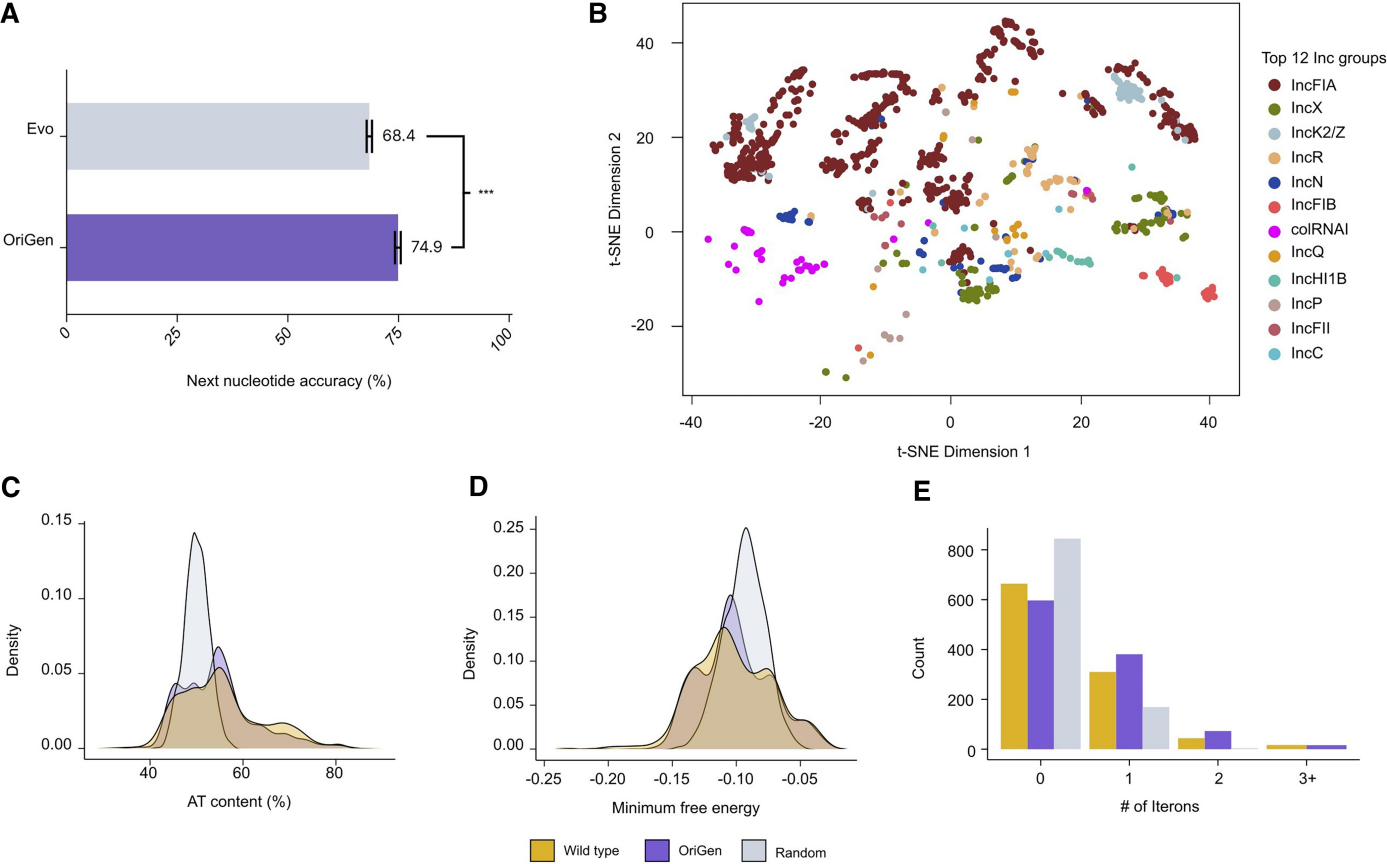
Evaluation and analysis of OriGen. (**A**) Next-nucleotide prediction accuracy comparison (two-sided bootstrap test, *P* < 0.001). (**B**) t-SNE plot of model embeddings for the top 12 incompatibility groups. (**C−E**), Analysis based on 1000 *oriVs* from unprompted model-generated replicons compared to equal numbers of sampled wild-type *oriVs* and random sequences, showing distributions of: (**C**), AT content, (**D**), minimum free energy, and (**E**), number of iterons.

As an initial validation, we evaluated OriGen’s ability to predict the next nucleotide in *oriV* sequences from our held-out IMG/PR test set. To ensure no near-duplicates of our training set were in our test set, we excluded any sequences with greater than 95% similarity (BLAST pident > 95%, qcovs > 95%) to those in our training data. We compared OriGen’s performance to Evo, which has recently emerged as a prominent genomic language model. Unlike OriGen, Evo was trained on IMG/PR itself, making direct comparison difficult. Moreover, Evo’s train/test splits were unavailable at the time of analysis, so a substantial portion of our evaluation set is likely part of Evo’s training data. Despite this considerable disadvantage, OriGen achieved significantly higher accuracy in predicting *oriV* sequences (75% versus 68%, Fig. 2A; bootstrap 95% CIs: [74.2−75.5] versus [67.8−69.0]). This performance gap suggests that specialized models can outperform general foundation models for specific biological tasks like replicon generation.

In this vein, we also tested whether OriGen would benefit from further specialization. Because plasmid origins fall into incompatibility groups—families that share similar replication and partitioning mechanisms, preventing plasmids from the same group from stably coexisting within a single cell—we trained new versions of the model using data from a single incompatibility group at a time. We then evaluated next-nucleotide prediction of these specialized models on held-out origins from the same incompatibility groups and compared their performance to the full OriGen model. For incompatibility groups that were well represented in the training data, the specialized and general models performed comparably. However, for groups with fewer examples, the specialized models performed much worse, while the full OriGen model maintained high accuracy. This indicates that OriGen is able to draw on information across groups and meaningfully generalize across different types of replicons (Supplemental Fig. 2).

Next, we explored whether OriGen’s learned replicon representations matched existing biological understanding of these regions. For each replicon, we averaged the per-token embeddings across its sequence to obtain a single vector representation. These representations were then projected into two dimensions using t-SNE. Coloring the resulting points by their incompatibility group revealed distinct and coherent clusters (Fig. 2B), suggesting that the model organizes replicons in a way that aligns with known biological classifications.

Beyond next-nucleotide prediction and embeddings, we also analyzed the characteristics of unprompted model-generated replicon sequences to assess their biological plausibility. We generated 1,000 sequences with no prompt (i.e. no specified species or Rep) and compared their *oriV* regions to those from wild-type plasmids and random sequences. While natural *oriV* sequences are diverse and not fully characterized, they often contain recognizable features such as AT-rich regions, low minimum free energy, and repeated sequences called iterons, which facilitate DNA replication initiation. The distribution of these motifs in our generated sequences closely matched that of wild-type *oriVs*, while random sequences showed markedly different patterns (Fig. 2C−E, Materials and methods). Additionally, we built a phylogenetic tree of the generated and wild-type origins together, revealing that model-generated *oriVs* are distributed throughout all major branches alongside wild-type *oriVs* (Supplemental Fig. 3). This indicates that the model generates diverse sequences rather than variations of a single template.

### Experimental validation

**Figure 3.**
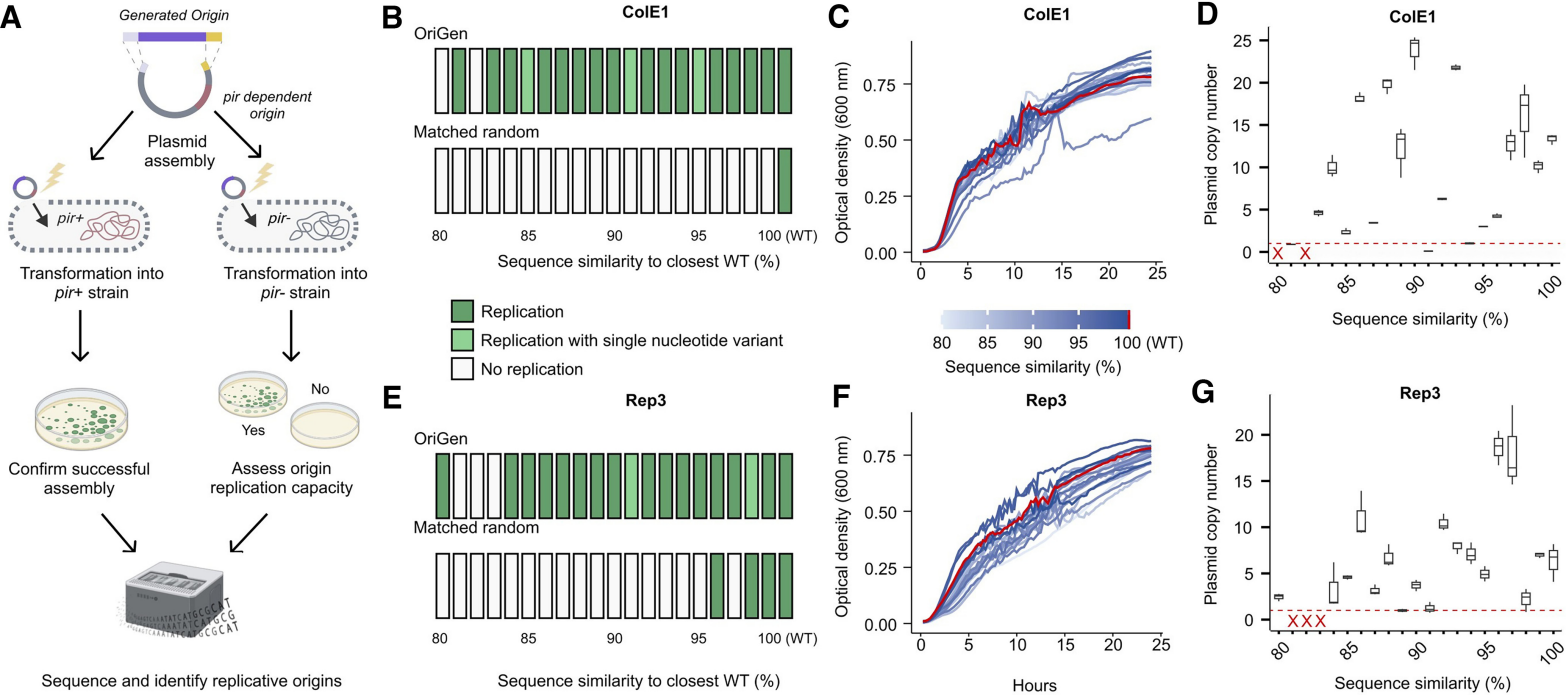
Functional validation of OriGen-generated origins. (**A**) Experimental workflow for validating generated origins (created with BioRender.com; adapted from Hasham, S., 2025; https://BioRender.com/s01r744). (**B**) Assessment of ColE1-type origin function in *pir- E. coli* strains. Matched random sequences contain the same number of mutations relative to wild-type as their corresponding OriGen-generated sequences. Lighter green indicates a single nucleotide polymorphism or single nucleotide indel was detected after sequencing (each bar represents a single replicate). (**C**) Growth curves for each ColE1-generated origin (blue gradient, lighter to darker indicates lower to higher sequence similarity) and wild-type control (red) in *pir-* strains. Each line represents the mean of three biological replicates. (**D**) Relative plasmid copy number of ColE1-generated origins in *pir-* strains. Boxplots represent data from three biological replicates. Red X’s denote origin sequences that did not replicate. (**E**) Assessment of uncharacterized Rep3-type origin function in *pir- E. coli* strains. (**F**) Growth curves for Rep3 generated origins in *pir-* strains. (**G**)Relative plasmid copy number results for Rep3 origins in *pir-* strains.

To validate that OriGen generates functional replication sequences, we tested their performance in bacterial cells (Fig. 3A). To test the generated *oriVs*, we synthesized and assembled them into vectors (R6K) that are dependent on a host gene (*pir*) for replication. The resulting constructs were transformed in parallel into *pir+* (EC100D) and *pir-* (NEB 10-beta) strains. The *pir +* strain served as an assembly control, as these cells would maintain the plasmid via the *R6K* origin regardless of the synthetic origin’s functionality. In contrast, growth in the *pir-* strain should only occur if our generated origin functioned. We confirmed the identity and integrity of constructs through full plasmid sequencing, allowing us to confirm the validity of the experiment and detect any mutations that arose during synthesis, assembly, or replication, which may indicate a “near miss” sequence that is one nucleotide away from being functional.

For our first experimental test, we focused on ColE1-type replicons, which are among the most thoroughly studied plasmid replication systems [35]. ColE1 origins include two overlapping antisense RNAs [36, 37], and typically function without a plasmid-encoded Rep protein. We prompted OriGen with an *E. coli* species token, no Rep protein sequence, and a 20-base seed sequence from a randomly selected ColE1 origin in our validation set. From 100,000 generated origins, we sampled candidates with varying levels of similarity to their closest matching wild-type origin in the training data. For each percent similarity bucket spanning from 80% to 99% we selected a single origin for experimental validation. We found that these generated sequences enabled plasmid replication across multiple similarity levels, with successful replication observed at similarities as low as 81% (Fig. 3B). In several cases (at 95%, 91%, and 85% similarity), sequencing revealed single base pair changes in the replicated plasmids. To test whether our success reflected genuine functional design rather than robustness to mutation, we introduced matched numbers of random mutations into the wild-type origins most closely related to the generated sequence. None of these controls enabled replication, a result that was highly significant (*p* < 0.001, one-sided Fisher’s exact test), suggesting that OriGen learned to preserve essential functional elements even in a replication system highly sensitive to mutations.

To evaluate OriGen’s performance beyond well-characterized replicons, we tested its ability to generate origins for a naturally occurring but previously uncharacterized plasmid. We identified an *E. coli* plasmid with no documented laboratory use and prompted OriGen with its Rep protein (Rep3) sequence to generate compatible *oriVs*. Following the same experimental pipeline as with ColE1, we cloned these generated origins alongside the Rep gene into our R6K testing system. We again observed successful replication across multiple similarity levels, with generated sequences functioning down to 80% similarity to known origins, while matched random controls did not function below 96% similarity (Fig. 3E, *p* < 0.001, one-sided Fisher’s exact test).

To move beyond a binary growth readout, we measured replicon function with several other quantitative assays comparing generated and wild-type origins. Plate reader measurements were used to evaluate the growth dynamics of *pir-* cells carrying origins under antibiotic selection. Generated origins showed growth comparable to wild-type controls, and growth rate did not vary with sequence similarity to the wild type (ANOVA with Tukey’s post hoc test and Spearman correlation yielding BH-adjusted p > 0.05; Fig. 3C and F; Supplemental Figs 4–5; Supplemental Table 2). We also quantified the relative copy number of plasmids containing generated and wild-type origins (see Methods). Copy number varied considerably across both origin types, but showed no significant decrease with sequence divergence from generated origins (Spearman correlation, BH-adjusted, *p* > 0.05, Supplemental Table 2, Fig. 3D and G).

We next assessed plasmid stability by measuring loss rates after 24 h of growth with or without antibiotic selection. Rep3-derived plasmids were considerably less stable than ColE1-derived plasmids: without selection, only 2 of 18 Rep3 variants retained >50% of plasmids on average compared to 16 of 19 ColE1 variants (Supplemental Fig. 6). This result suggests that plasmids with Rep3 origins may require additional plasmid-encoded proteins to maintain stable replication in the absence of selective pressure. However, across all origins, plasmid stability was not significantly correlated with similarity to wild types (Spearman correlation, BH-adjusted, p > 0.05, Supplemental Table 2).

Finally, since most ColE1 origins remained stable over 24 h, we next assessed fitness over longer timescales using pooled competition experiments to determine if more subtle differences in plasmid fitness emerged. Wild-type and generated variants were passaged for three consecutive 24-h intervals with and without antibiotic selection. At each time point, we sequenced the origins to track changes in variant frequency and calculated log_2_ relative fitness scores (Methods). With or without selection, the majority of the origins exhibited near neutral fitness (Supplemental Fig. 7). As expected, the origins that were most unstable in the curing assay (sequence similarities: 81%, 91%, and 94%) also had strong negative fitness scores (<−1) and/or went extinct in both treatments. Taken together, generated origins performed comparably to WT controls in all experiments, and sequence dissimilarity from WT did not predict function.

### Analysis of functional characteristics

**Figure 4.**
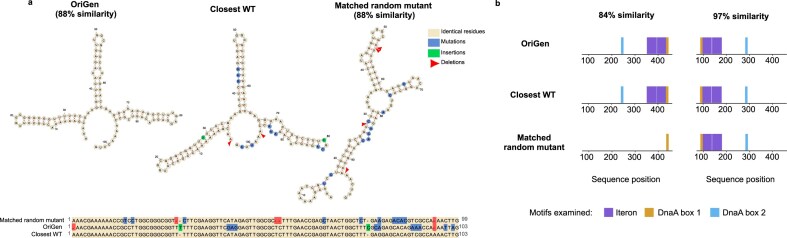
Functional characteristics of OriGen sequences. (**A**) RNAI secondary structure comparison of one OriGen-generated ColE1 example versus the closest wild type (WT) versus the matched random mutant control. Mutations (blue), insertions (green), and deletions (red) relative to the WT are highlighted. (**B**) Distribution of known motifs in examples of Rep3 OriGen-generated origins versus closest WT versus matched random mutant. In both examples, the model-generated origins led to replication while the random mutants did not.

To understand how OriGen preserved functionality while generating novel sequences, we examined trios of related sequences: model-generated origins, their nearest wild-type matches, and corresponding randomly mutated versions. For the ColE1 trios, we analyzed RNAI secondary structures, a well-characterized regulatory element [38, 39] that forms a cloverleaf structure that inhibits ColE1 replication, providing copy number control [40] (Fig. 4A). Generated sequences generally maintained their characteristic secondary structure despite multiple nucleotide differences from their wild-type counterparts. In contrast, in the matching randomly mutated sequences, similar numbers of differences typically disrupted the cloverleaf structure (Supplemental Fig. 8). Despite training only on DNA sequences, OriGen learned to navigate the inherent structural sensitivity of origins by strategically introducing variations that preserve critical functional features.

Analysis of Rep3 sequence trios revealed patterns in the conservation of known functional motifs (Fig. 4B). In most cases, matched random mutations disrupted essential elements like iterons and DnaA boxes, while OriGen preserved them, directly explaining the differences in functionality. More intriguingly, in another case, both the generated and randomly mutated sequences maintained all known motifs, yet only the model-generated sequence enabled replication (97% similarity sequences, Fig. 4B, Supplemental Fig. 9 for all sequences). This suggests that beyond the currently characterized motifs, there are additional sequence features essential for replication that our model learned to preserve. OriGen’s ability to maintain these uncharacterized but crucial elements, even while generating substantially different sequences, demonstrates the potential for AI systems to capture complex biological design constraints that extend beyond our current understanding.

Interestingly, while the generated ColE1 sequences differed from associated wild types in positions scattered throughout the origin, the variations in the Rep3 generated origins were more concentrated in the terminal 60 bp at the 3' end of the sequence (Supplemental Fig. 10). The model seems to be signaling a non-essential region of the Rep3 origin, suggesting its ability to be used for origin boundary detection. To validate this, we removed the terminal 60 bp from a wild-type Rep3 origin and found that the plasmid replicated (Extended Supplementary Data S4).

### Constraint-guided generation

To explore whether OriGen could be directed toward practical bioengineering objectives, we applied a restriction-site-avoidance task, with the goal of producing functional ColE1-type origins lacking any instances of the most common *E. coli* restriction sites (Methods). These sites are significant hurdles to genetic engineering, as they can decrease plasmid transformation efficiencies by many orders of magnitude when recognized by the target bacteria [41−43]. Among wild-type ColE1 origins, this list of restriction sites occurred a median of seven times per origin, and all natural origins over 400bp had at least one restriction site in their sequence (Supplemental Fig. 11).

**Figure 5.**
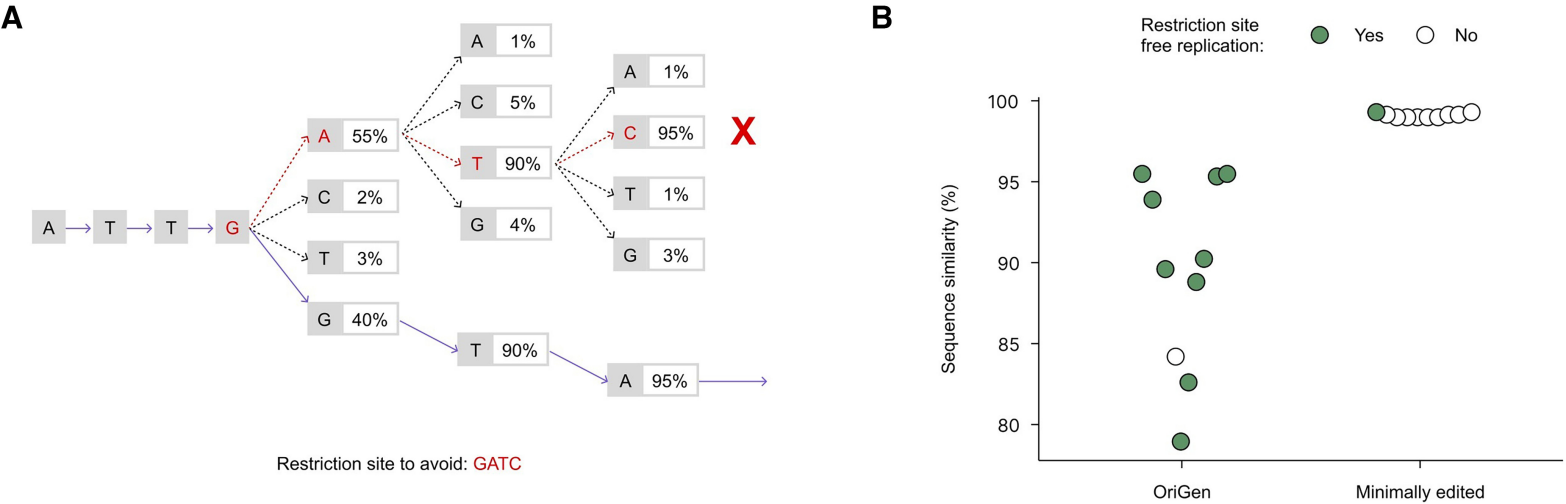
Constraint-guided generation of restriction-site-free ColE1 origins. (**A**) Schematic of constraint-guided generation showing beam search exploration of sequence possibilities. At each position, the model computes probabilities for different options (branches) and maintains several candidate sequences simultaneously, allowing it to identify better paths that might require earlier divergence from the most probable route. Red X indicates a path containing a forbidden restriction site motif, and purple arrows indicate the model’s chosen path. (**B**) Experimental validation comparing OriGen-generated restriction-site-free origins with minimally modified wild-type origins. The *y*-axis indicates sequence similarity to the nearest wild type. Green circles indicate that the origin yielded replication.

Using the same ColE1 seed prompt, we directed the model to generate origins free of these restriction sites by providing a list of forbidden sequence motifs during generation (Fig. 5A). This constraint-guided approach uses beam search to explore multiple sequence possibilities simultaneously, allowing the model to identify alternative paths that avoid problematic motifs. For comparison, we also modified a wild-type ColE1 origin by manually removing all occurrences of these sites via minimal random substitutions (Materials and methods).

Experimental validation revealed that only one of the ten minimally edited wild-type origins yielded replication, despite diverging by only a handful of randomly substituted base pairs (Fig. 5B, Supplemental Table 3). For the OriGen designs, despite significantly lower similarity to a wild-type origin, we found that nine of the ten generated origins successfully supported replication (*P* < 0.001, one-sided Fisher’s exact test). These results come with one caveat: all recovered plasmids carried a single additional mutation relative to the intended sequence. Interestingly, the positions of mutations differed for each sequence, and different single mutations also occurred in the *pir +* strain assembly controls. These mutations potentially suggest that the initial designs imposed a replication burden, such as an overly high copy number, that favored adaptive change. Nevertheless, the mutations did not reintroduce any of the restriction sites, yielding replicating, restriction-site-free origins. Together, these results provide proof-of-principle that OriGen can generate biologically functional plasmid origins outside the distribution of natural sequences while still meeting user-defined sequence constraints.

## Discussion

In this work, we have developed and experimentally validated a language model capable of generating functional origins of replication, including designs that satisfy explicit user-defined sequence constraints. By assembling a comprehensive dataset of plasmid replicons and their bacterial hosts, we trained a model that learned to preserve the complex requirements for plasmid replication while exploring variations in sequence. Our experimental validation in *E. coli* across two fundamentally different replication mechanisms, combined with proof-of-principle demonstrations of constraint-guided design, highlights OriGen’s potential as a flexible tool for engineering-specific biological sequences.

While this work demonstrates promising results, several limitations should be noted. Our training dataset, while substantially larger than previous collections, captures only a subset of replicon diversity. This constraint stems from our computational pipeline, which necessarily restricts us to sequences similar to documented origins in DoriC, likely missing undiscovered replication mechanisms present in nature. Our experimental validation centered on *E. coli* as a model system; though our architecture incorporates host information, testing host-specificity across diverse microbes requires further model development and remains as future work. Furthermore, subtler impacts of generated origins, which may have been missed by copy number, growth, and curing assays, could be observed by transcriptomic profiling and assaying replication under stress challenges. Finally, like all machine learning systems, OriGen generates sequences derived from patterns in its training data, producing novelty from combinations and variations of natural sequence elements, rather than inventing entirely new mechanisms.

Despite these caveats, this work represents the first demonstration of AI-generated sequences capable of biological replication. As with other advances in AI-guided sequence design, including generation of proteins [44, 45], regulatory and coding DNA [46–49], and functional RNAs [50, 51], these results highlight both new opportunities and dual-use concerns. In the present case, the generated plasmid origins remain closely aligned with natural replicons and fall well within the bounds of long-established laboratory practice, comparable to outcomes from rational engineering or directed evolution [39, 52]. Looking forward, however, advances in model capability may extend beyond close variants of natural sequences, making it timely to discuss ethics and governance of AI-driven design, considering the current lack of regulatory frameworks [53, 54].

The ability to generate functional replication computationally enables new opportunities for biological design. This capability opens the door to designing plasmid components optimized for diverse engineering goals, from avoidance of defense system recognition sequences to size minimization. OriGen also opens the path to the creation of origins functional in diverse bacterial hosts, which is of particular interest for the broad majority of bacteria that lack robust genetic tools. Extending OriGen’s capabilities to whole plasmid generation could facilitate targeted microbiome editing across clinical, industrial, and agricultural applications. This would allow researchers to quickly move from sequencing the community to design, synthesis, and deployment of host-compatible plasmids functionally tailored to address undesirable microbiome characteristics. While significant technical hurdles remain to achieve this vision, OriGen represents a step toward programming biological replication for human-defined purposes.

## Supplementary Material

gkaf1198_Supplemental_Files

## Data Availability

Source code for model training, inference, and evaluation is available on GitHub (https://github.com/j-irvine/origen) and figshare (https://figshare.com/articles/software/origen/28578281?file=52931255). The repository includes scripts for sequence generation, evaluation metrics, alignment utilities, and example notebooks demonstrating usage and the sequence similarity analysis pipeline. Pre-trained models and dataset files are publicly hosted on HuggingFace (https://huggingface.co/jirvine). Sequences of experimentally validated origins are provided in the supplementary information. Experimental results and analyses are available on FigShare (https://figshare.com/articles/dataset/OriGen_experimental_data/30246868).
