# Supplementary material for: Generating functional plasmid origins with OriGen
Source: Nucleic Acids Res. 2025 Nov 29;53(22):gkaf1198. doi: 10.1093/nar/gkaf1198 (PMC12663086; doi:10.1093/nar/gkaf1198)
Supplement: gkaf1198_Supplemental_Files [file gkaf1198_supplemental_files.zip › Supplemental Figures (NAR Revision) (4).pdf]

Supplemental Figures

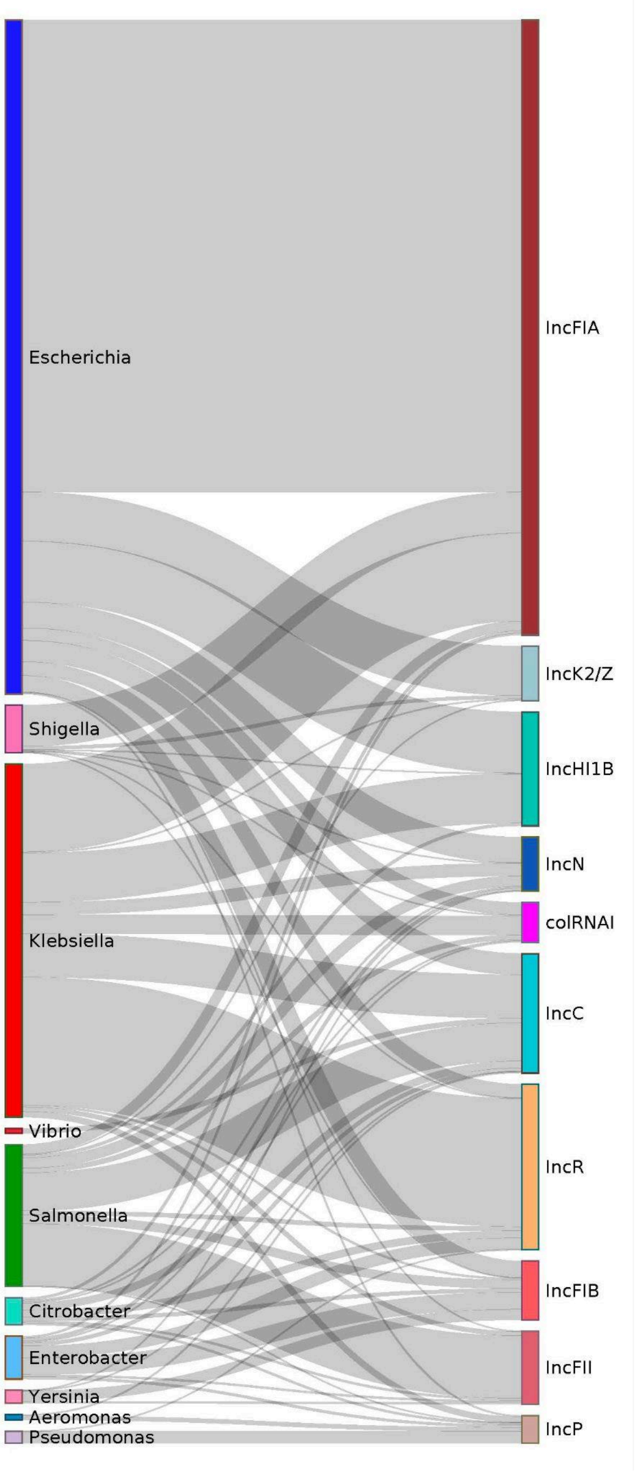

Supplemental Figure 1: Distribution of host species and incompatibility groups in the OriGen training and validation combined dataset. Sankey plot showing the relationship between bacterial host species (left) and the top 10 incompatibility groups (right) for replicons in the combined DoriC and PLSDB data.

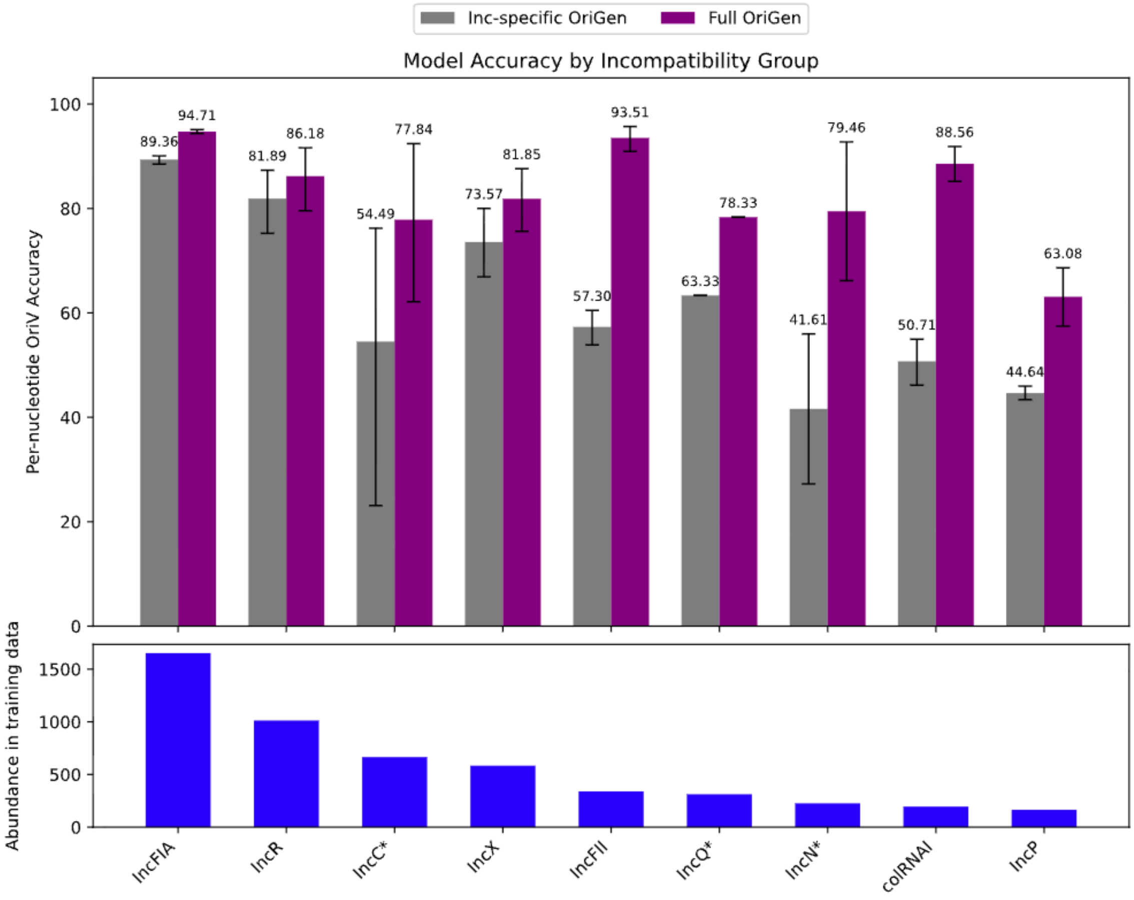

Supplemental Figure 2: Comparison of specialized versus general OriGen models across incompatibility groups. Per-nucleotide prediction accuracy for incompatibility group-specific models (gray bars) versus the full OriGen model (purple bars), with training data abundance shown below (blue bars). Evaluation performed on held-out oriV sequences from IMG/PR. Error bars represent 95% confidence intervals estimated by bootstrapping (n=1,000 resamples). Asterisks indicate incompatibility groups with fewer than 10 sequences in the held-out evaluation set. Three of the top 12 incompatibility groups (IncF1B, IncK2/Z, and IncHI1B) were excluded from this analysis due to lack of representation in the held-out evaluation set.

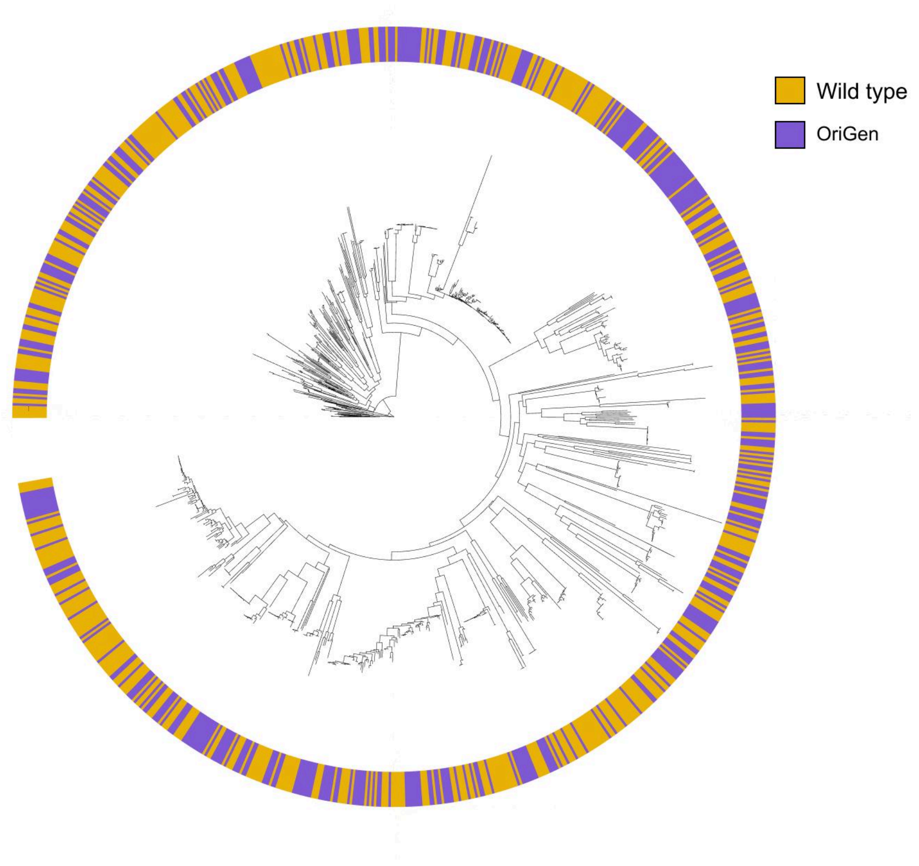

Supplemental Figure 3: Phylogenetic tree of model-generations vs wild types. A phylogenetic tree of the generated and wild-type origins were generated using Fastree using General Time Reversible (GTR) substitution model. This reveals that model-generated oriVs are distributed throughout all major branches alongside wild-type oriVs.

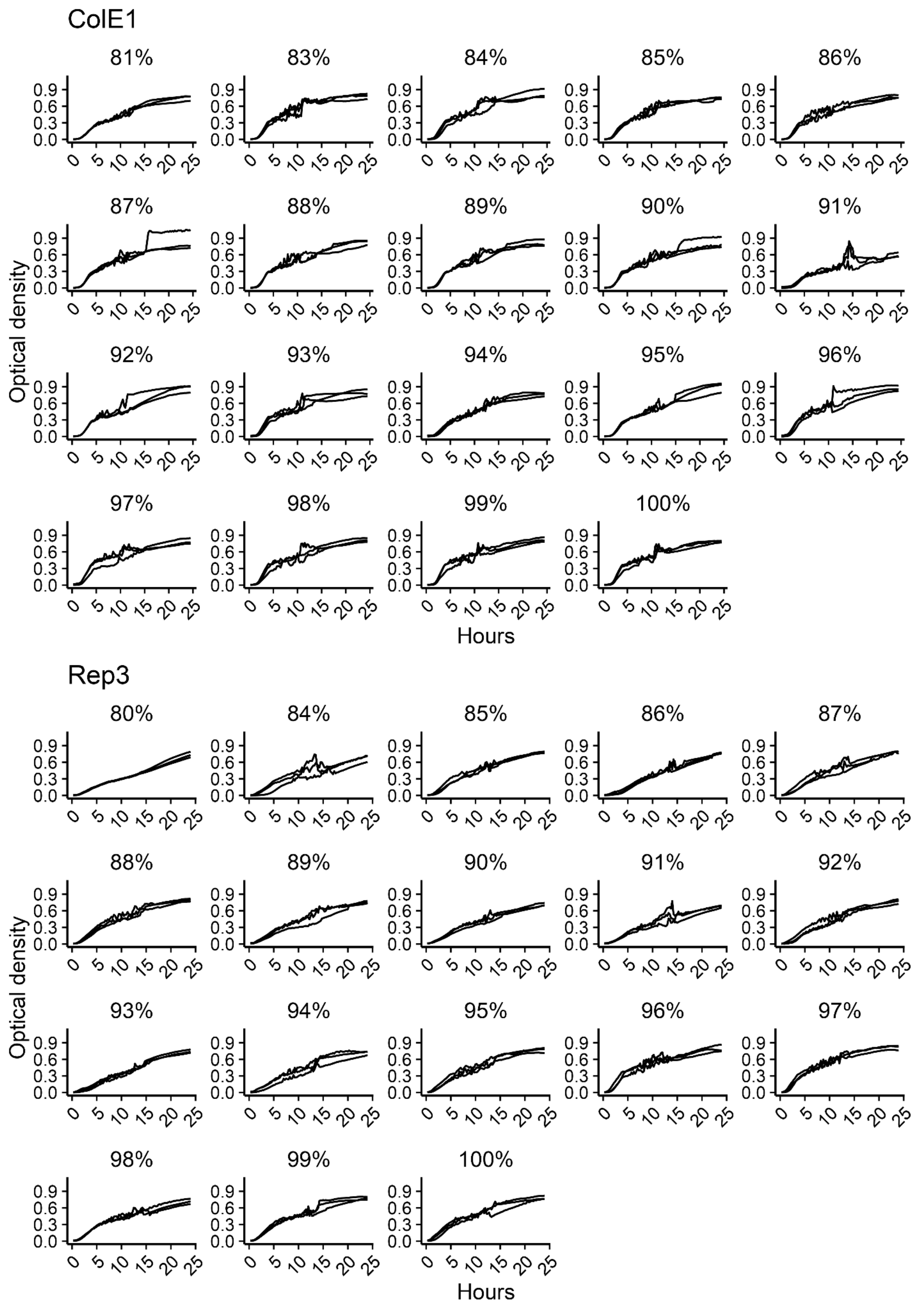

Supplemental Figure 4: Individual growth curves for each origin. Growth curves (optical density 600_{nm}) for ColE1 (top) and Rep3 (bottom) wild type and generated origins. Each line represents the blank subtracted optical growth curve of an individual biological replicate. Growth curves are faceted by sequence similarity.

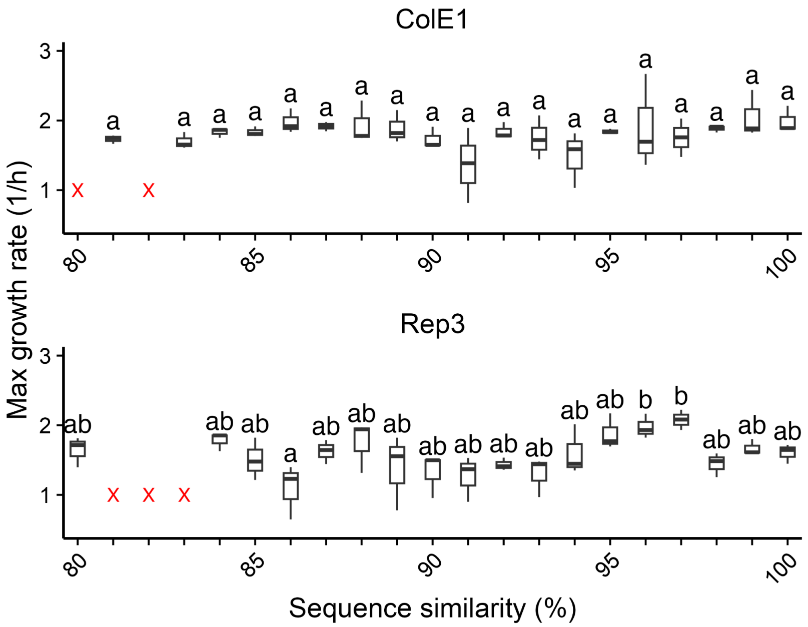

Supplemental Figure 5: Growth rates of cells bearing wild type and generated origins. Growth rates for cells bearing ColE1(top) and Rep3 (bottom) plasmids in media with antibiotic selection. Each box represents three biological replicates. One way ANOVAs were performed separately for ColE1 and Rep3 origins with sequence similarity as the factor followed by Tukey’s post-hoc test. Letters above boxes indicate statistical groupings: origins that share a letter are not significantly different, while those with different letters differ at $p < 0.05$. Red “X” symbols denote generated origins that failed to replicate and were excluded from this analysis.

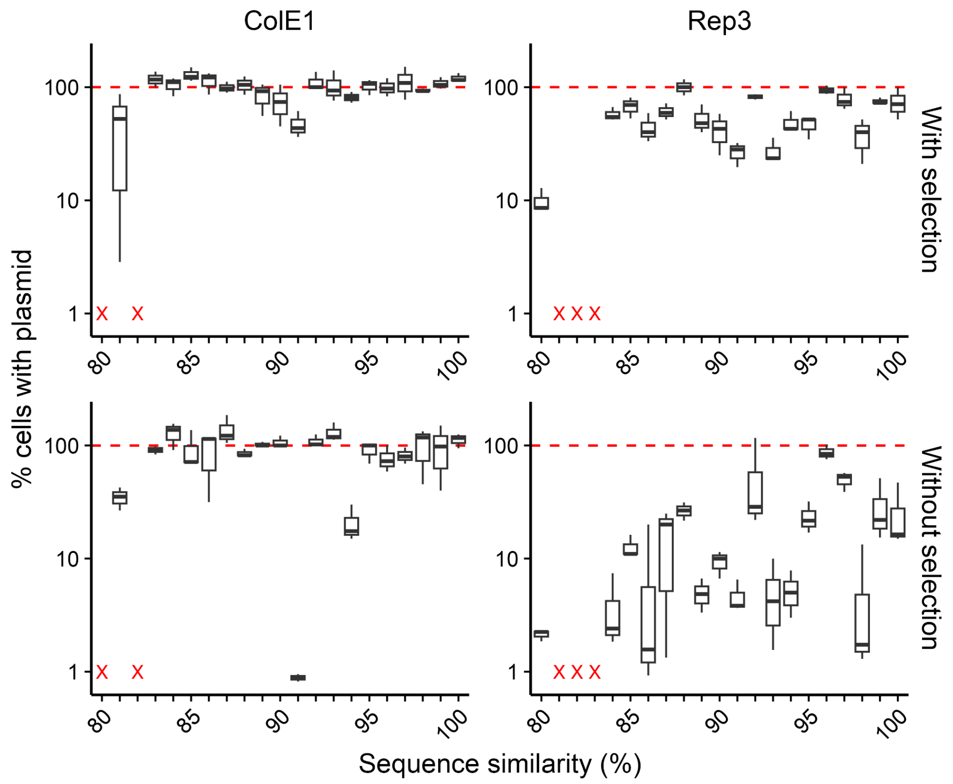

Supplemental Figure 6: Plasmid retention in cells bearing wild type and generated origins. Percent of cells with plasmids bearing ColE1 (left column) and Rep3 (right column) origins after growth in media with (top row) or without (bottom row) antibiotic selection. Each box represents three biological replicates. The y-axis is shown on a $\log_{10}$ scale. Red “X” symbols denote generated origins that failed to replicate and were excluded from this analysis.

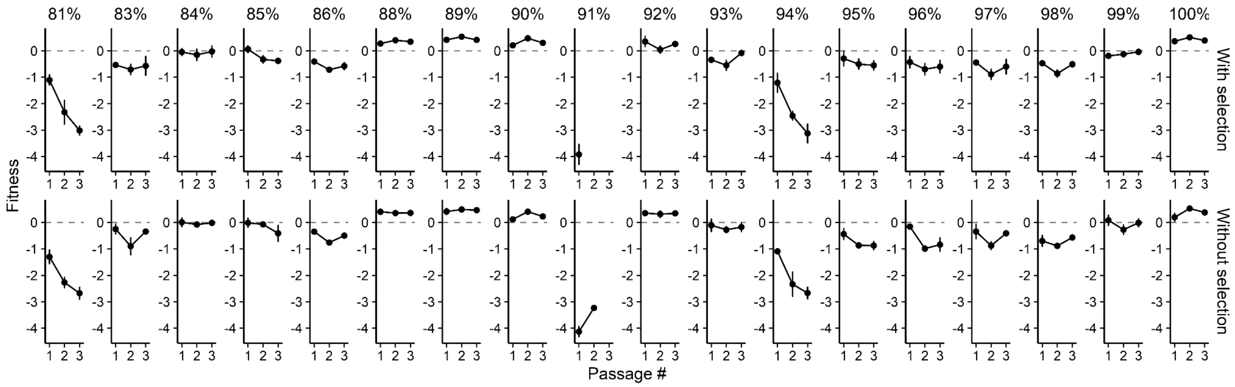

Supplemental Figure 7: Fitness trajectory of ColE1 origins in pooled competition assay. Relative fitness scores of origins in media with antibiotic selection (top) and without selection (bottom) measured over three consecutive 24 hour passages. Each column represents a unique origin faceted by its percent sequence similarity to wild-type. The y-axis shows the relative fitness score calculated as the log_2-transformed ratio of a variants frequency at each passage relative to its starting frequency (time 0). Each point represents the mean fitness from three biological replicates, and error bars indicate the standard error of the mean. The dashed line at y=0 indicates neutral fitness. Points were not plotted for variants after they became undetectable.

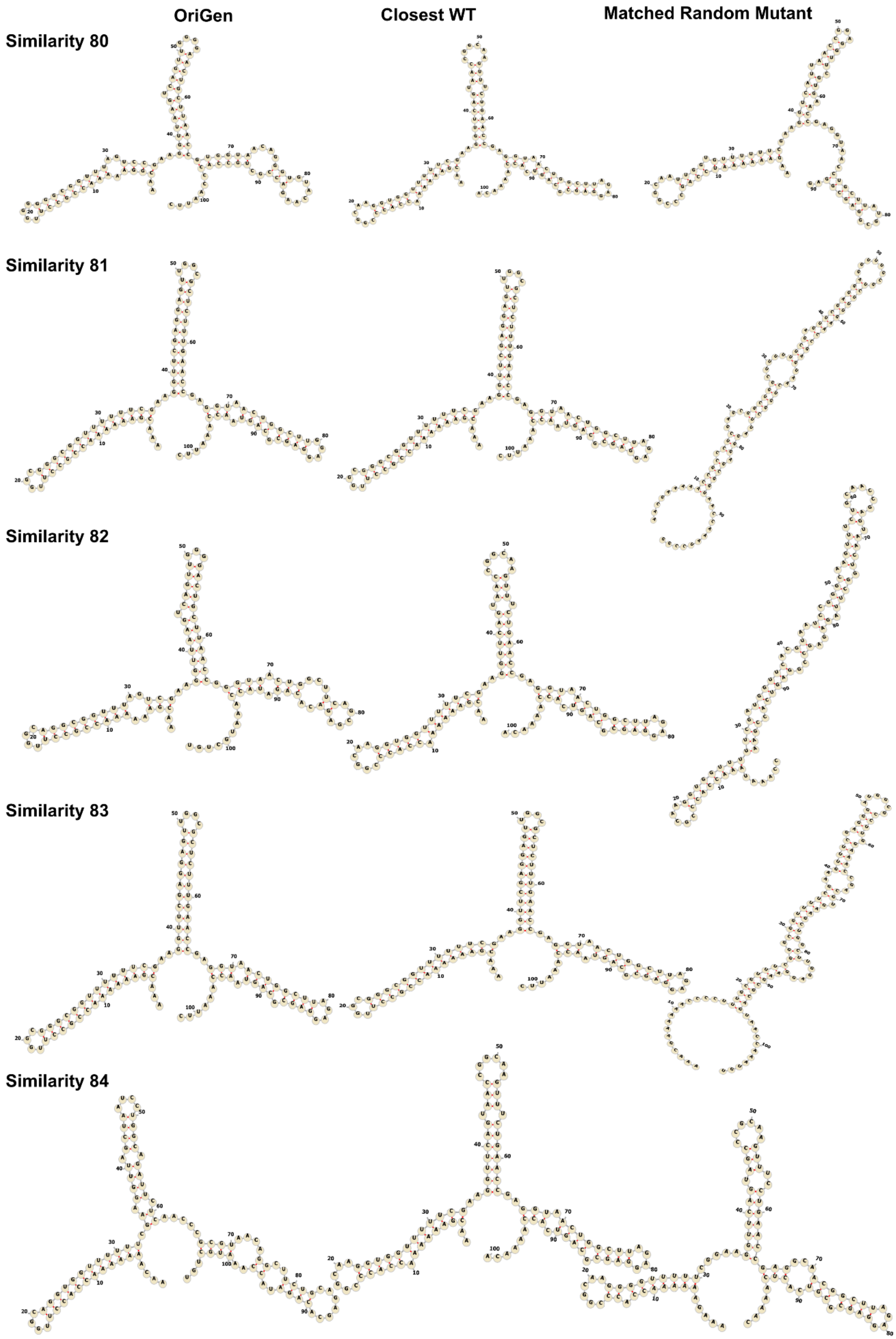
OriGen
Closest WT
Matched Random Mutant
Similarity 80
Similarity 81
Similarity 82
Similarity 83
Similarity 84

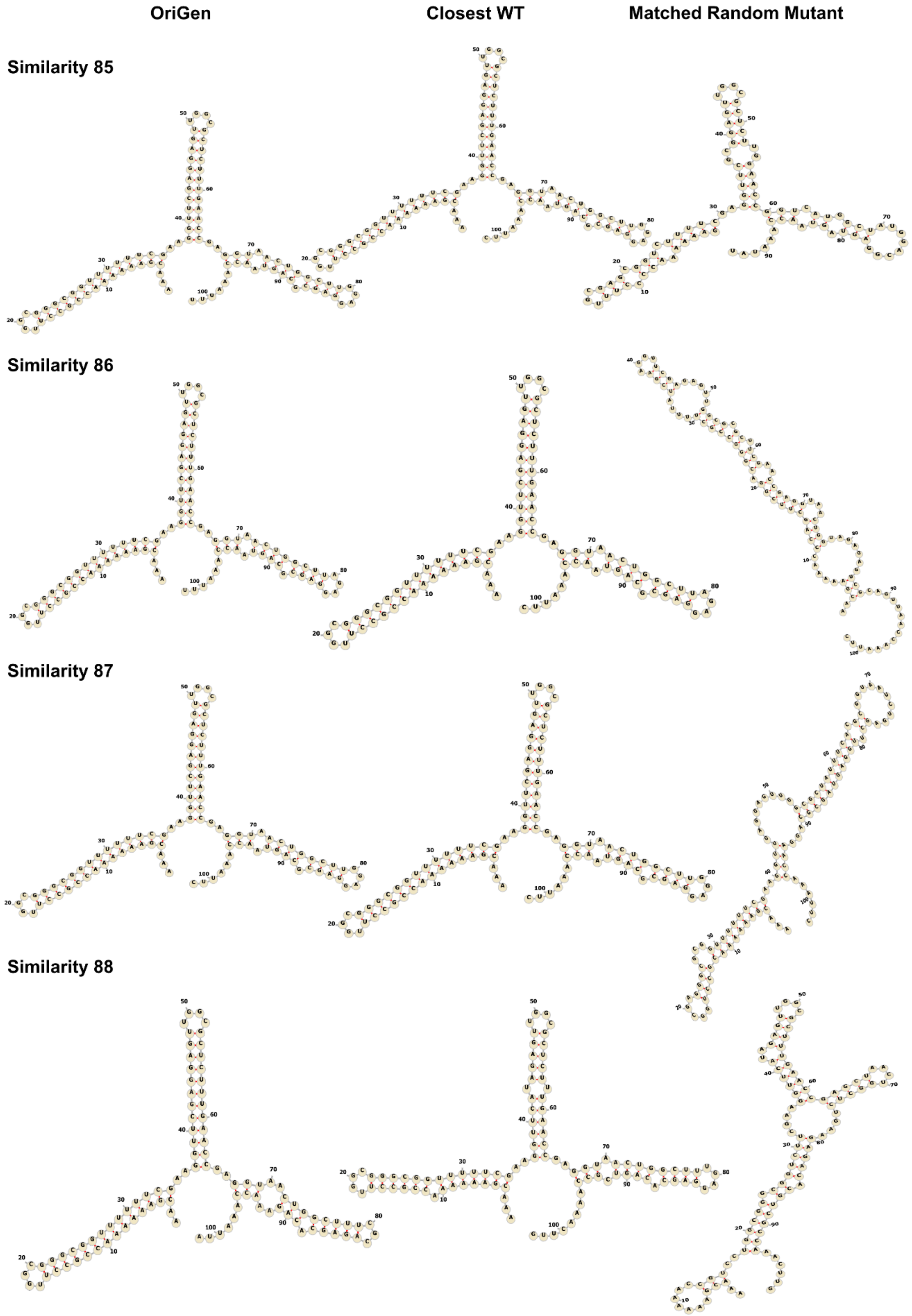
OriGen
Closest WT
Matched Random Mutant
Similarity 85
Similarity 86
Similarity 87
Similarity 88

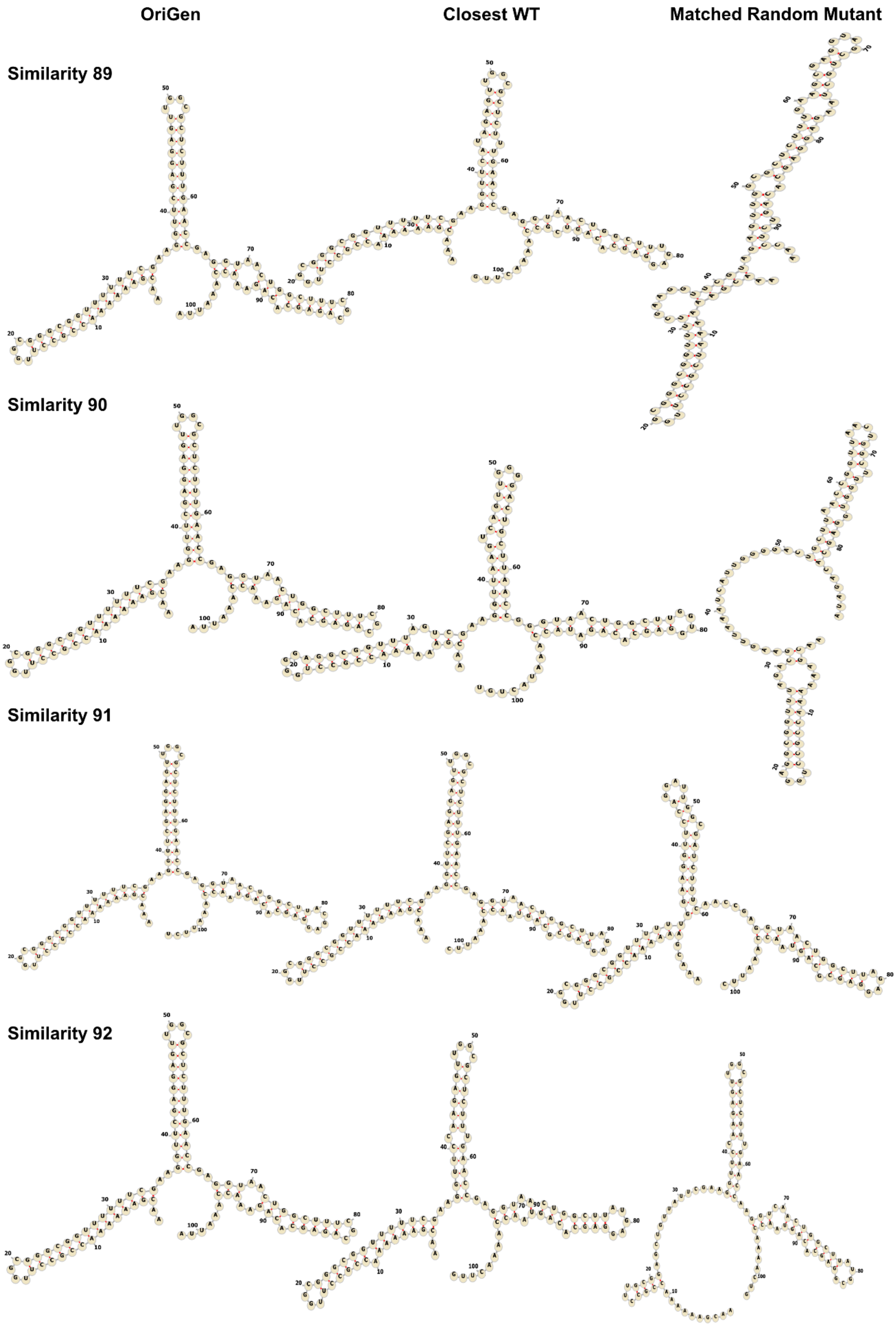
OriGen
Closest WT
Matched Random Mutant
Similarity 89
Simlarity 90
Similarity 91
Similarity 92

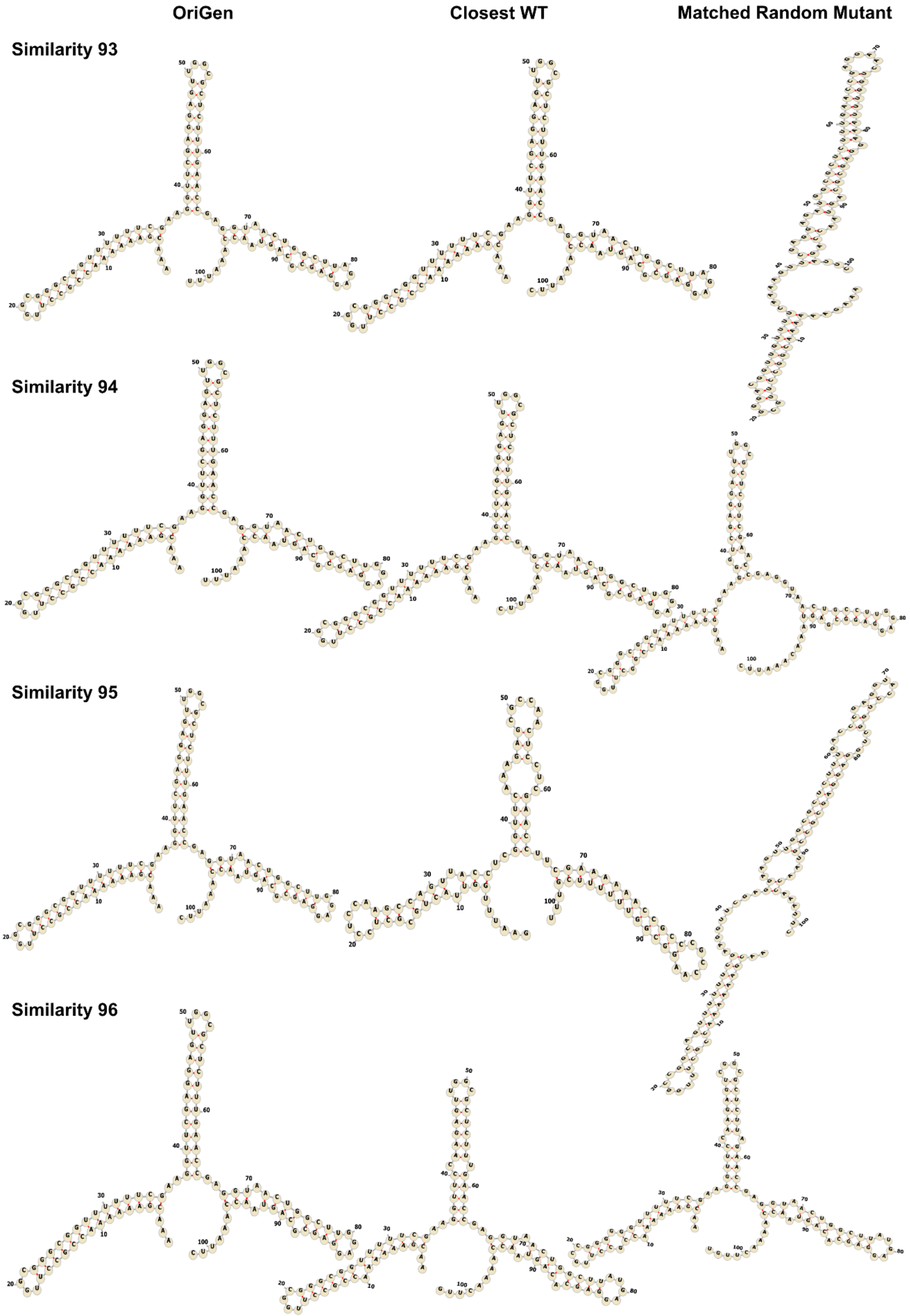
OriGen
Closest WT
Matched Random Mutant
Similarity 93
Similarity 94
Similarity 95
Similarity 96

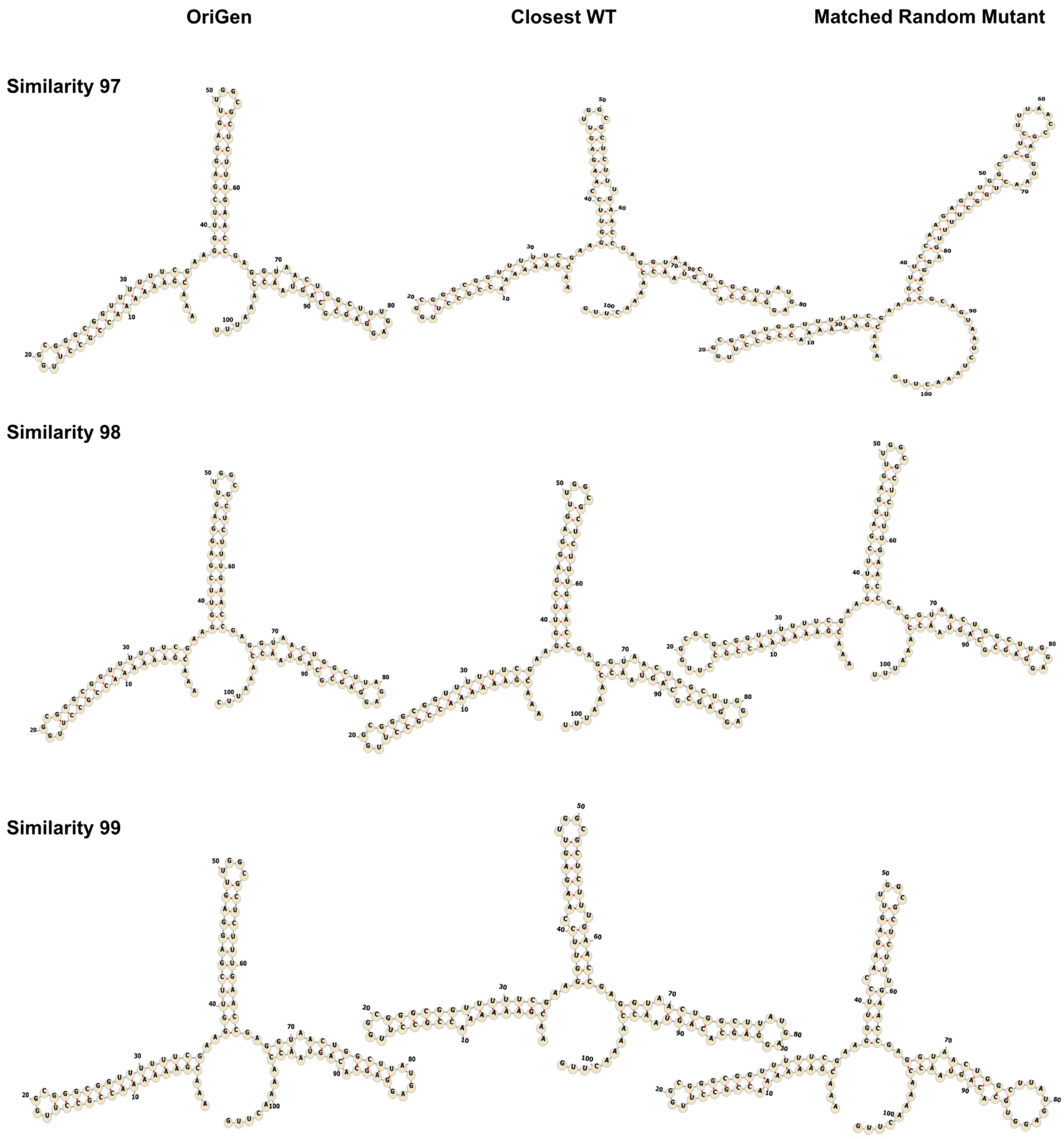

Supplemental Figure 8: Predicted structures of ColE1 RNAI for the closest wild type (WT) origin, OriGen generated, and matched random mutant controls. Structures are arranged by sequence similarity to the full wild-type origin sequence, as determined using the Needleman-Wunsch algorithm.

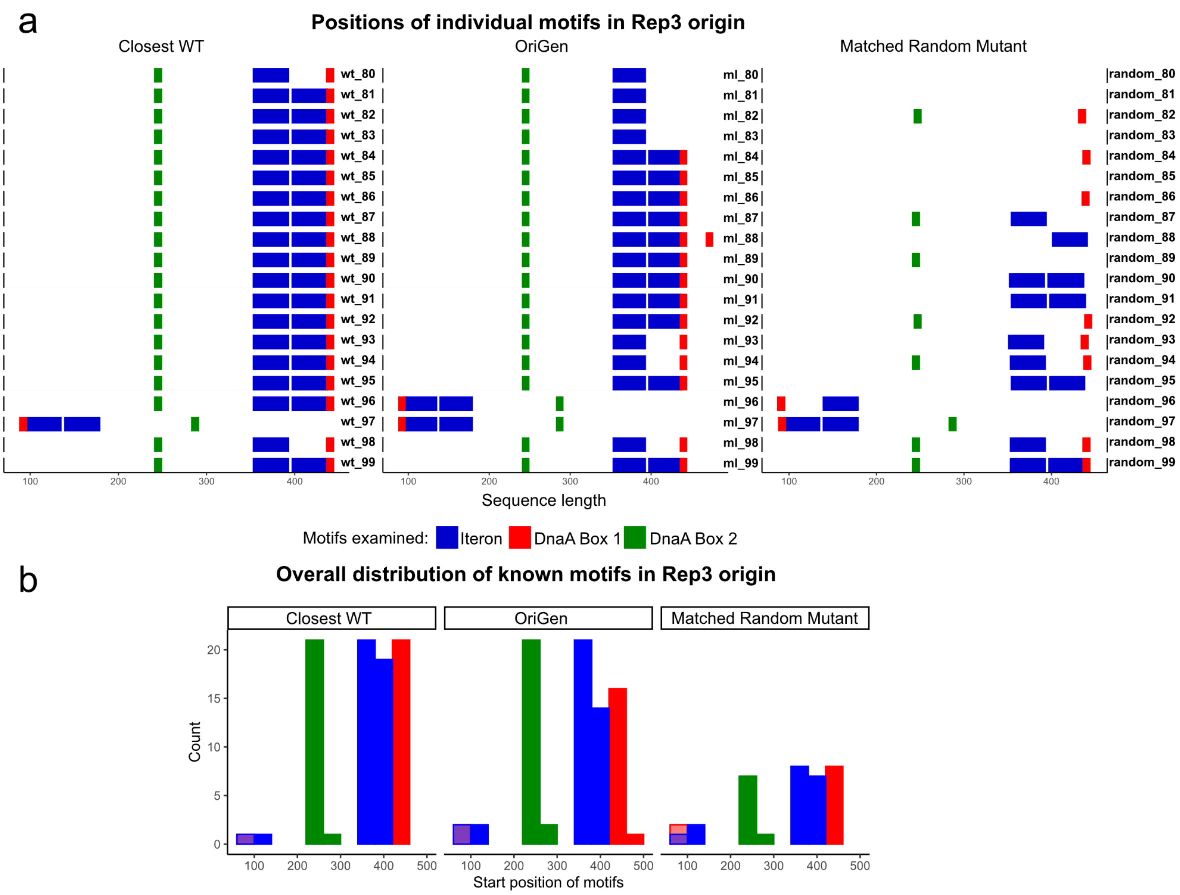

Supplemental Figure 9: Detection of motifs in Rep3 origins. a, Known motifs identified in individual origin sequences ordered by Needleman-Wunsch similarity. **b,** Overall counts of motifs identified in the closest wild-type (WT) origin, OriGen generated, and matched random mutant controls. Iteron motifs are colored blue, DnaA box 1 is red, DnaA box 2 is green.

a

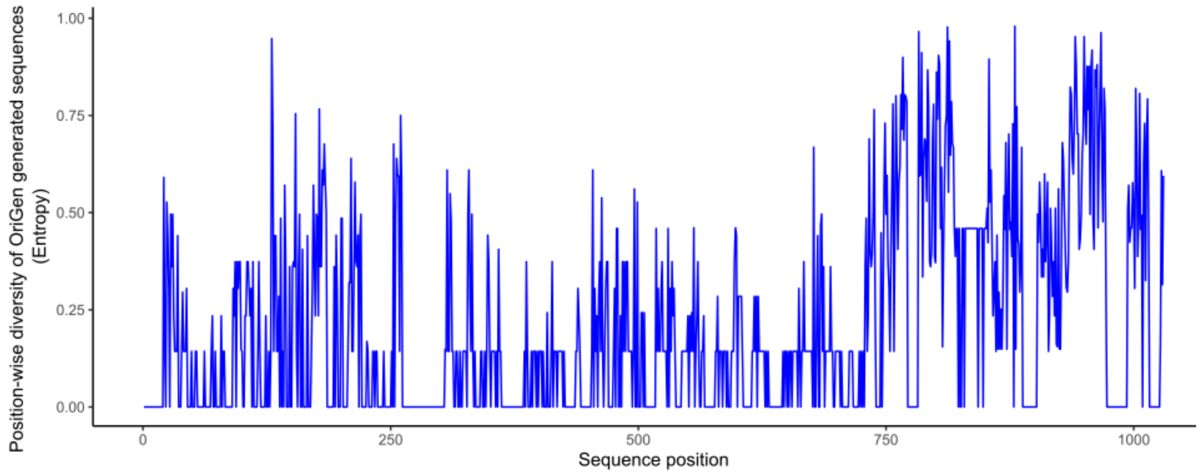

b

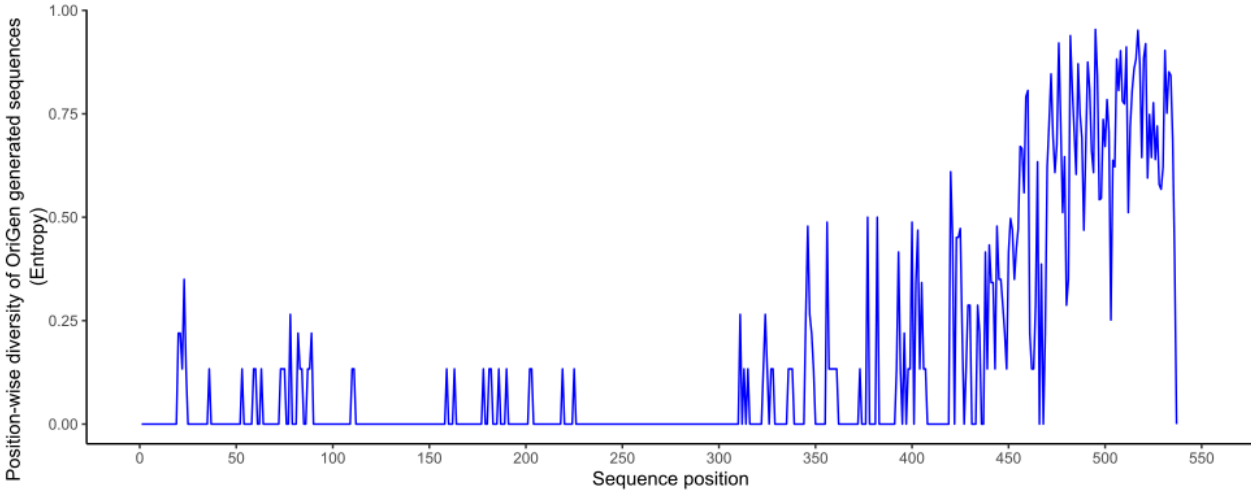

Supplemental Figure 10: Sequence entropy analysis of OriGen-generated origins. a, Position-wise Shannon entropy plot of aligned OriGen-generated ColE1-type origins **b,** Corresponding analysis for Rep3-type origins. Lower entropy values indicate higher conservation across sequences.

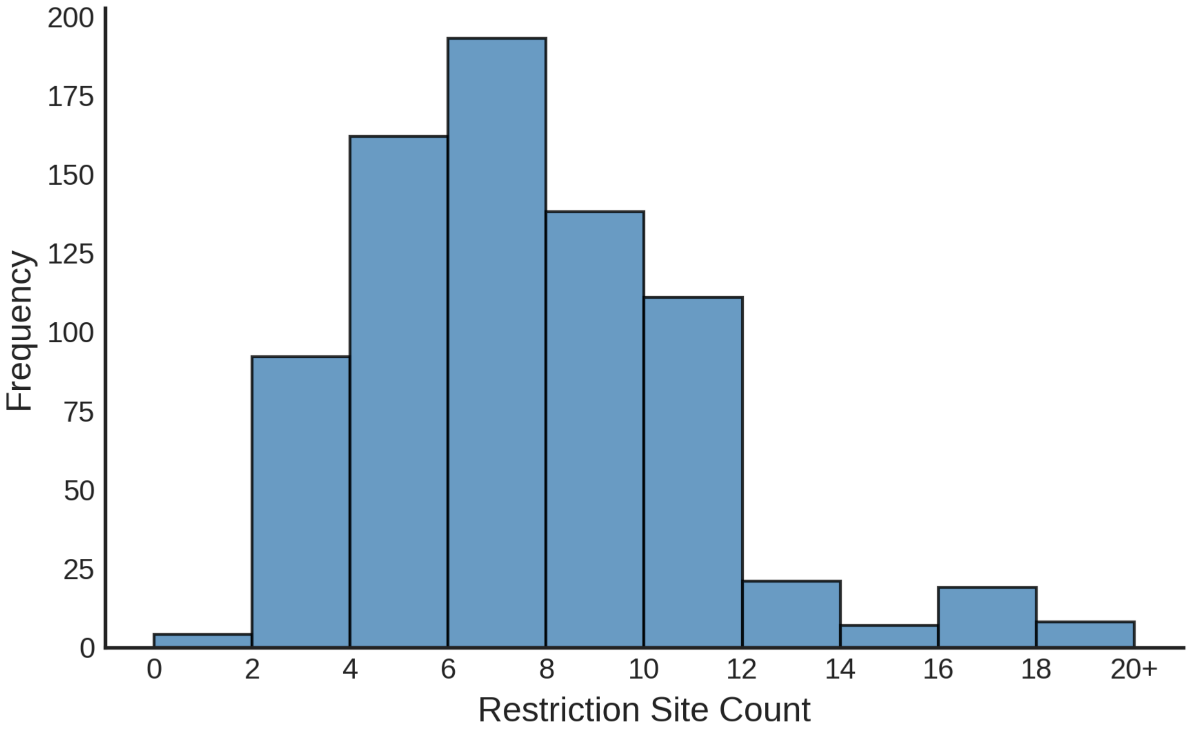

Supplementary Figure 11: Distribution of number of restriction sites in wild-type ColE1 origins. Histogram showing the number of common *E. coli* restriction sites per ColE1 origin of replication in the training dataset. Restriction sites analyzed include the twelve most frequent type II restriction-modification recognition sites in *E. coli*: GATC, CCWGG, GGTCTC, GAAABCC, GRGCYC, CCGCGG, CTGCAG, CCNGG, GGYRCC, CRARCAG, GAATTC, and CCTNAGG. Median = 7 sites per origin

Origin type	Similarity bracket	OriGen BLAST pident	OriGen BLAST query coverage	OriGen Needleman % similarity	Random Needleman % similarity
ColE1	80	95.19	69.71	80.28	81.42
ColE1	81	95.39	79.95	80.83	81.88
ColE1	82	95.41	70.11	81.88	82.97
ColE1	83	97.37	77.76	82.93	83.31
ColE1	84	96.84	77.76	83.8	85.19
ColE1	85	96.62	76.81	84.82	85.24
ColE1	86	97.31	81.17	85.6	85.73
ColE1	87	99.12	77.76	87.08	87.02
ColE1	88	92.07	95.53	87.64	88.27
ColE1	89	92.24	94.41	88.61	88.71
ColE1	90	91.16	100.61	90	90.08
ColE1	91	91.06	100.68	90.92	90.79
ColE1	92	92.7	100.68	91.98	91.98
ColE1	93	93.2	100.27	92.55	92.55
ColE1	94	94.17	100.55	94.05	94.05
ColE1	95	94.7	100.41	94.58	94.72
ColE1	96	96.47	100.14	96.47	96.61
ColE1	97	96.74	100.14	96.74	96.74
ColE1	98	97.96	100	97.96	97.96
ColE1	99	98.64	100.14	98.64	98.64
Rep3	80	91.1	90.79	80.59	81.75
Rep3	81	100	78.72	81.91	82.12
Rep3	82	100	78.72	82.02	82.59
Rep3	83	98.45	84.77	83.98	83.78
Rep3	84	98.66	84.56	84.86	86.02
Rep3	85	99.78	84.56	85.74	86.07
Rep3	86	98.08	88.14	86.94	87.45
Rep3	87	99.34	85.31	87.88	87.77
Rep3	88	98.91	86.44	88.47	88.79
Rep3	89	98.46	85.5	89.45	90.15
Rep3	90	98.29	87.97	90.71	91.54
Rep3	91	96.94	92.29	91.56	91.87

Rep3	92	96.78	93.42	92.72	92.9
Rep3	93	96.83	94.92	93.51	94.05
Rep3	94	96.89	96.8	94.59	94.59
Rep3	95	96.2	99.06	95.15	95.34
Rep3	96	96.8	100.19	96.8	96.8
Rep3	97	97.36	100	97.36	97.36
Rep3	98	98.87	100	98.87	98.87
Rep3	99	99.81	100	99.81	99.81

Supplemental Table 1: Detailed sequence similarity metrics for experimentally tested origins. Alignment and BLAST metrics for OriGen-generated sequences and matched random mutant controls. Each row shows the origin type, similarity bracket, BLAST percent identity (pident) and query coverage of the top hit when comparing OriGen-generated sequences against the training dataset, Needleman-Wunsch global alignment similarity score between OriGen-generated sequences and their best matching wild-type sequence identified by BLAST, and Needleman-Wunsch similarity score between random mutant controls and the same wild-type sequence. Random mutants contain identical numbers and types of mutations as their OriGen-generated counterparts but with random positional distribution.

Origin type	Predictor	Response	n	Pseudo R2	P-value	BH adjusted P-value
ColE1	Sequence similarity	Growth rate	19	0.330	0.168	0.672
ColE1	Sequence similarity	Plasmid copy number	19	0.232	0.340	0.680
ColE1	Sequence similarity	Plasmid retention with selection	19	0.154	0.529	0.705
ColE1	Sequence similarity	Plasmid retention without selection	19	0.029	0.907	0.907
Rep3	Sequence similarity	Growth rate	18	0.193	0.443	0.443
Rep3	Sequence similarity	Plasmid copy number	18	0.346	0.160	0.320
Rep3	Sequence similarity	Plasmid retention with selection	18	0.247	0.324	0.432
Rep3	Sequence similarity	Plasmid retention without selection	18	0.525	0.025	0.101

Supplemental Table 2: Spearman correlations measuring the relationship between origin sequence similarity and experimental variables. For each origin type (ColE1 and Rep3), % sequence similarity relative to WT sequences was tested as a predictor or maximum growth rate, relative plasmid copy number, and % of plasmids retained after 24 hours of growth (with or without antibiotic selection). Values exceeding 100% plasmid retention were capped at 100%, as such values were attributable to pipetting error. Response variables represent the average of three biological replicates for each origin type. Sample size (n), pseudo-R^2, unadjusted p-values, and Benjamini-Hochberg (BH) adjusted p-values are reported. No correlations remained significant after multiple comparisons correction.

**Restriction-site-free sequences**	**Sequence similarity to nearest WT**	**Replication in *pir* -?**
Random substitution origin 1	98.8	No
Random substitution origin 2	98.8	No
Random substitution origin 3	98.9	No
Random substitution origin 4	98.9	No
Random substitution origin 5	98.9	No
Random substitution origin 6	99.0	No
Random substitution origin 7	99.0	No
Random substitution origin 8	99.0	No
Random substitution origin 9	99.2	Yes, with 1 bp SNP
Random substitution origin 10	99.2	No
OriGen-designed origin 1	78.8	Yes, with 1 bp insertion
OriGen-designed origin 2	82.4	Yes, with 1 bp SNP
OriGen-designed origin 3	84.0	No
OriGen-designed origin 4	88.6	Yes, with 1 bp SNP
OriGen-designed origin 5	89.5	Yes, with 1 bp SNP
OriGen-designed origin 6	90.1	Yes, with 1 bp SNP
OriGen-designed origin 7	93.8	Yes, with 1 bp SNP
OriGen-designed origin 8	95.2	Yes, with 1 bp deletion
OriGen-designed origin 9	95.3	Yes, with 1 bp insertion
OriGen-designed origin 10	95.4	Yes, with 1 bp SNP

Supplemental Table 3: Functional testing of wild-type ColE1 origins with restriction sites substituted via minimal random mutation (first 10 rows) and OriGen-generated ColE1 origins designed to avoid restriction sites.
